# Pathogen delivery route impacts disease severity in experimental *Mycoplasma ovipneumoniae* infection of domestic lambs

**DOI:** 10.1186/s13567-024-01439-y

**Published:** 2025-01-13

**Authors:** Bryan Tegner Jacobson, Jessica DeWit-Dibbert, LaShae Zanca, Sobha Sonar, Carol Hardy, Michael Throolin, Patricia C. Brewster, Kaitlyn Andujo, Kerri Jones, Jonathon Sago, Stephen Smith, Lizabeth Bowen, Diane Bimczok

**Affiliations:** 1 Department of Microbiology and Cell Biology, Montana State University, Bozeman, MT USA; 2 Department of Mathematical Sciences, Montana State University, Bozeman, MT USA; 3 Anatomic and Clinical Pathology, Histology, and Milk Laboratory, Montana Veterinary Diagnostic Laboratory, Bozeman, MT USA; 4 United States Geological Survey, Davis Field Station, University of California, Davis, CA USA

**Keywords:** *Mycoplasma ovipneumoniae*, sheep, pneumonia, delivery route

## Abstract

*M. ovipneumoniae* is a respiratory pathogen that can cause mild to moderate pneumonia and reduced productivity in domestic lambs. However, studies on both natural and experimental *M. ovipneumoniae* infection have reported highly variable clinical signs and pathology. Here, we assessed the impact of administering *M. ovipneumoniae* to the upper respiratory tract (URT) or to the lower respiratory tract (LRT) of two-month-old specific pathogen-free lambs. Lambs were inoculated with PBS (control) or with ceftiofur-treated nasal wash fluid obtained from sheep with natural *M. ovipneumoniae* infection, monitored for eight weeks, and subsequently euthanized. All lambs in the URT and LRT groups developed a stable infection with *M. ovipneumoniae*. *M. ovipneumoniae* infection led to lower weight gains and mild respiratory disease, with significantly greater effects following LRT inoculation compared to URT inoculation. At necropsy, lambs inoculated via the LRT showed consolidation of the cranial lung lobes. In addition, histological signs of  alveolar, bronchiolar, and interstitial inflammation were significantly more severe in the LRT compared to the URT group. *M. ovipneumoniae* loads in the trachea and bronchi also were significantly higher after LRT than URT inoculation. Interestingly, 9/10 inoculated lambs also tested positive for *M. haemolytica* in nasal swab but not in bronchial swab samples. In summary, our study suggests that bypassing protective mechanisms of the URT by delivering respiratory pathogens to the LRT leads to more severe respiratory disease and lung damage than delivery to the URT.

**Supplementary Information:**

The online version contains supplementary material available at 10.1186/s13567-024-01439-y.

## Introduction

*Mycoplasma ovipneumoniae* is a respiratory pathogen of domestic and wild sheep that is associated with atypical pneumonia and decreased productivity in lambs [1–3] and that is highly common across the world [4–8]. However, results from experimental *M. ovipneumoniae* infections have been inconsistent, ranging from severe respiratory illnesses with weight loss and behavioral changes to asymptomatic infections [3, 9–14]. These variable results have been partially attributed to the presence or absence of bacterial and viral co-infections [11, 15–17], differences in the virulence of *M. ovipneumoniae* strains [3, 18], and genetic differences in host susceptibility [19]. Current evidence from infection experiments and observational studies point to two potential outcomes of *M. ovipneumoniae* infection: asymptomatic carrier status, with colonization of the upper airways and no clinical disease [14, 20–22], or clinical infection with atypical pneumonia, where *M. ovipneumoniae* is found in diseased lung tissue [12, 14, 22, 23]. While clinical pneumonia is generally an immediate concern, sheep that are asymptomatic carriers for *M. ovipneumoniae* commonly remain undetected. However, these animals play an important epidemiological role, since they can spread infection to susceptible lambs and cause pneumonia outbreaks [24, 25]. Despite these two disparate outcomes, how *M. ovipneumoniae* distribution in the respiratory tract impacts *M. ovipneumoniae* pathogenesis has not yet been systematically analyzed.

The upper respiratory tract (URT), which is comprised of the nasal cavity, pharynx, and cranial portions of the larynx, is the primary site of infection for most respiratory pathogens [26]. Under natural circumstances, respiratory pathogens can reach the lower respiratory tract (LRT) if respiratory defenses such as the mucociliary elevator are damaged due to previous infections or environmental stressors, or in association with physical exercise and exertion [27]. Natural *M. ovipneumoniae* infection is thought to occur via respiratory droplets or secretions following repeated close contact [3], but airborne transmission over a distance of > 10 m also has been reported [12]. Upon initial infection, *M. ovipneumoniae* can damage the cilia of the respiratory epithelium [28], which then enables the *M. ovipneumoniae* as well as other pathogens to invade the lower respiratory tract. Ionas et al. proposed that different *M. ovipneumoniae* strains might preferentially colonize the lungs versus the nasal passages [29].

We hypothesized that infection of the lower respiratory tract, achieved experimentally through intratracheal inoculation, would result in clinical disease, whereas pathogen delivery to the upper respiratory tract would result in asymptomatic colonization. We present results from an experimental infection study performed in specific pathogen free (SPF) domestic lambs that were inoculated via either the URT or the LRT with antibiotic-treated nasal wash fluid from lambs naturally infected with *M. ovipneumoniae*. Our results indicate that delivering the infectious inoculum directly to the LRT resulted in increased pathogen load in the trachea and bronchi, decreased weight gains, increased clinical signs, and increased gross and microscopic respiratory tract pathology compared to pathogen delivery to the nasal and oral mucosae. Interestingly, both URT and LRT delivery of *M. ovipneumoniae* enabled expansion of previously undetectable *Mannheimia haemolytica* in the upper respiratory tract of infected lambs from both groups, which may have contributed to the development of clinical disease in this study.

## Materials and methods

### Animals and husbandry conditions

Fifteen two- to three-month-old mixed-breed lambs (nine males, six females) from Montana State University’s (MSU’s) SPF flock [21, 30] were used in this study. The lambs were born naturally to SPF ewes, weaned at approximately 6 weeks of age, and kept in outdoor paddocks with ad libitum access to water, hay, mineral and selenium supplements, and lamb starter pellets. All lambs were vaccinated with a multivalent clostridial vaccine (Covexin® 8, Merck). One week before experimental infections took place, the lambs were moved to the Johnson Family Livestock Facility (JFLF) ABSL-2 laboratory, where they were housed in two separate heated animal rooms (15.5–16.8 °C). The animals were fed a standard diet consisting of hay, lamb concentrate, and starter pellets. Clean water, mineral, salt, and selenium supplements were provided ad libitum. All animal care personnel changed into sterile personal protective equipment prior to entering the animal rooms, and personnel responsible for the lambs did not have any contact with other non-SPF sheep for the duration of the experiment. All animal experiments in this study were approved by the Institutional Animal Care and Use Committee (IACUC) of MSU, protocol #2022-158-95.

### Preparation of inocula

Bacterial inocula containing *M. ovipneumoniae* were prepared from fresh nasal wash fluids of fifteen lambs naturally infected with respiratory pathogens, as described previously [21]. Briefly, *M. ovipneumoniae-*infected lambs at MSU’s Red Bluff Research Ranch were selected based on prior positive tests results from nasal swab samples. Nasal washes were performed by squirting a total of 60 mL of sterile PBS into the lambs’ nostrils and collecting the flush fluid into clean, single use polyethylene bags (ZipLock®). The nasal wash fluids were pooled, diluted with an equal amount of tryptic soy broth, incubated for 2 h at 37 °C, and then concentrated by centrifugation at 10 000 × *g* for 30 min. Inocula were prepared by resuspending pellets from equal amounts of starting material in 20 mL of sterile PBS per dose for LRT inoculation and 50 mL per dose for URT inoculation. Aliquots of the untreated and the ceftiofur-treated nasal wash were sent to the Washington Animal Diseases Diagnostic Laboratory for bacteriological analysis and *M. ovipneumoniae* PCR.

### Experimental design and sample collection

Fifteen experimental animals were randomly selected from a total of 22 SPF lambs born in our flock in 2022 and randomly assigned to three treatment groups consisting of two female and three male animals: (1) uninfected control; (2) upper respiratory tract (URT) infection with nasal wash fluid; and (3) lower respiratory tract (LRT) infection with nasal wash fluid. “Dam” was used as a blocking variable to ensure that siblings were placed in different groups. Random selection and random assignment was performed using the R package randomizr (v 0.22.0) [31]. Animal characteristics are provided in Table 1. There were no statistically significant differences in sex, litter size, starting weight or age. One week before the experimental start date (day -7), lambs were moved into two separate animal rooms (one for the control group (*n* = 5) and one for the URT and LRT groups (*n* = 10)) inside the JFLF. All infected lambs from the URT and the LRT groups were housed in the same room, with no barrier between the groups. Husbandry staff wore sterile scrubs, head and face coverings, and latex gloves when entering rooms and always handled the control group before entering the room with the URT and LRT groups. Lambs in the URT and LRT groups were inoculated with the infected nasal wash fluid, and control lambs received PBS. For URT infection, 30 mL of inoculum was administered into the lamb’s nostrils, 10 mL into the conjunctival sacs, and 10 mL into the oral cavity. For LRT infection, 20 mL of inoculum was administered directly into the distal trachea using a flexible fiber-optic endoscope measuring 6.6 mm × 100 cm (VFS-2B VetVu, Swiss Precision Products, Inc, Oxford, MA) under local anesthesia with lidocaine. Serum samples, heparinized blood samples, and PAXgene® tube (BD Biosciences) samples for RNA isolation were collected every two weeks by jugular venipuncture. Bronchoalveolar lavage fluid was collected at −1, 2, and 5 weeks pi (post-infection) using a fiber optic endoscope, and cell concentration was determined with a Neubauer counting chamber. At the experimental endpoint, all lambs were euthanized and transferred to the Montana Veterinary Diagnostic Laboratory (MVDL) for necropsy and tissue collection. One lamb in the URT group developed a severe urinary tract disease that did not respond to treatment and was therefore euthanized during week five of the study, and no endpoint samples were collected.

**Table 1:** **Characteristics of experimental animals**

Experimental group	N	Treatment	Route	Males	Litter size	Weight at start (kg)	Age at start (d)
Control	2	PBS	URT	3	2.2 ± 0.4	27.7 ± 9.1	64.0 ± 11.1
	3	PBS	LRT				
URT^1^	5 (4)^3^	Nasal wash	URT	3	1.6 ± 0.5	28.5 ± 4.5	59.8 ± 7.2
LRT^2^	5	Nasal wash	LRT	3	1.6 ± 0.9	31.2 ± 6.5	67.2 ± 8.6
*P* value				> 0.99	0.288	0.709	0.460

^1^Inoculation of upper respiratory tract via nasal, conjunctival, and oral mucosae.

^2^Inoculation of lower respiratory tract via endoscope.

^3^One lamb was euthanized prior to the experimental endpoint due to urinary tract disease. Groups were compared using one-way ANOVA with Tukey’s multiple comparisons test.

### Assessment of clinical signs and other animal characteristics

Following experimental *M. ovipneumoniae* infection or mock treatment, lamb health status was assessed twice daily by trained JFLF personnel, as previously described [21, 30]. Rectal temperatures were measured twice a week and whenever an animal showed any signs of disease. The following health parameters were assessed and were used to calculate a clinical disease score: (1) respiratory signs; (2) general behavior; (3) appetite, and (4) any medications administered exclusive of the experimental antibiotic treatment as detailed in Additional file 1. Daily scores represent the sum of the two values obtained for each day. Body temperatures were measured twice per week. Lamb body weights were determined weekly using a digital scale.

### Evaluation of pathological and histopathological changes

Sheep were necropsied at the MVDL within 30 min of euthanasia, and the respiratory tract including lungs and the pleural cavity were evaluated for pathological changes by trained veterinary pathologists (S.S. and J.S.). Representative hematoxylin/eosin-stained formalin-fixed paraffin-embedded sections of the cranial and caudal right and left lung lobes were scored for the presence of histopathological alterations. The scoring criteria were based on a publication by Passmore et al. [32], with slight modifications (see Table 2), and the pathologist was blinded to the experimental groups.

**Table 2:** **Scoring criteria for histopathological assessment of lung tissues, based on Passmore 2018, Respiratory Research** [32]

Score	Pulmonary edema and congestion	Alveolar and interstitial inflammation	Bronchiolar inflammation	BALT hyperplasia
0	Minimal/Normal	Minimal/Absent	Minimal/Normal	None
1	Mild edema and/or congestion	Mild inflammatory exudate	Mild infiltration of inflammatory cells	Mild
2	Moderate edema and/or congestion	Moderate inflammatory exudate	Moderate infiltration of inflammatory cells	Moderate
3	Severe edema and congestion ± intra-alveolar hemorrhage	Marked-severe inflammatory exudate	Marked/severe infiltration by inflammatory cells, ± epithelial damage	Marked

### *M. ovipneumoniae* serology

Analysis of serum samples for the presence of *M. ovipneumoniae-*reactive antibodies was performed at the Washington Animal Disease Diagnostic Laboratory (WADDL) using the laboratory’s competitive enzyme-linked immunosorbent assay (cELISA) test, which has a diagnostic sensitivity of 88% and a diagnostic specificity of 99.3%. Data are shown as % inhibition and represent the reduction in binding of the labelled monoclonal antibody to the *M. ovipneumoniae* test antigen caused by competitive binding of serum antibodies from the diagnostic samples.

### Detection of *M. ovipneumoniae* and *Pasteurellaceae*

Flocked polyester swabs (Puritan) were stored at −20 °C in transport medium composed of DMEM with 15% glycerol, and aliquots were shipped on dry ice to WADDL. Standard microbiological assays, performed at WADDL, were used to detect and identify *Pasteurellacea*. A subset of samples that tested positive for *M. haemolytica* also was tested for the presence of leukotoxin by PCR [33]. *M. ovipneumoniae* was detected by quantitative PCR (qPCR) using WADDL’s standard diagnostic assay. In addition, qPCR detection of *M. haemolytica* was performed in our laboratory using a protocol previously described by Shanthalingam et al. [33]. Primer sequences are provided in Table 3. All PCR data are expressed as the 40 -cT value of the sample.

**Table 3:** **List of primers**

Target	Sequence (5’-3’)	Product size	References
*M. ovipneumoniae* p113		295 bp	[21, 60]
Forward	GGG GTG CGC AAC ATT AGT TA		
Reverse	CTT ACT GCT GCC TCC CGT AG		
*M. haemolytica* o-sialoglycoprotein endopeptidase		227 bp	[33]
Forward	TGG GCA ATA CGA ACT ACT CGG G		
Reverse	CTT TAA TCG TAT TCG CAG		
*M. haemolytica* Leukotoxin (*lktA*)		497 bp	[33]
Forward	CTT ACA TTT TAG CCC AAC GTG		
Reverse	TAA ATT CGC AAG ATA ACG GG		

### Analysis of cytokine and inflammatory marker gene expression

Whole blood collected into PAXgene® tubes was processed for RNA isolation and qRT-PCR analysis as previously described [34]. Lung tissue collected at the experimental endpoint from two regions of the cranial lung and two regions of the caudal lungs was frozen at −80 °C and stored until analysis. Total RNA was extracted from homogenized tissue using the RNeasy Lipid Tissue Mini Kit (Qiagen; Germantown, MD, USA). To remove contaminating genomic (g)DNA, the spin columns were treated with RNase-free DNase I (DNase, Amersham Pharmacia Biotech Inc.; PA, USA) at 20 °C for 15 min. cDNA synthesis was performed on 2 µg of RNA template from each tissue. Reaction conditions included 4 U reverse transcriptase (Omniscript, Qiagen, Valencia, CA, USA), 1 µM random hexamers, 0.5 mM of each dNTP, and 10 U RNase inhibitor, in RT buffer (Qiagen, Valencia, CA, USA). Reactions were incubated for 60 min at 37 °C, followed by an enzyme inactivation step of 5 min at 93 °C, and then stored at −20 °C until further analysis. Expression of the following genes was measured: aryl hydrocarbon receptor (*AHR*), heat shock protein 70 (*HSP70*), myeloid differentiation primary response 88 (*MYD88*), myxovirus resistance-1 (*MX1*), interferon-γ (*IFNG*), interleukin-1β (*IL1B*), *IL10*, *CD69*, Fas-associated death domain (*FADD*), AMP-activated protein kinase (*AMPK*), tumor necrosis factor-α (*TNFA*), GATA binding protein 3 (*GATA3*), T-box expressed in T cells (*TBX21*), and transforming growth factor-β (TGFB). Primer sequences are provided in [34]. Data were analyzed using the delta-delta Ct method (2^−ΔΔCt^), with S9 used as housekeeping gene. Gene expression for each animal was normalized to values obtained immediately prior to inoculation (week 0) for blood samples, or to the average gene expression of the control group for tissue samples.

### Statistical analyses

Data were analyzed using R Statistical Software (v4.3.3; R Core Team 2024) [35] and GraphPad Prism 10.1.2 (Boston, MA) and are shown as mean ± SD unless stated otherwise. Data for the total health score, percent weight gain, and temperature models was processed into data structures with the tidyverse package (v2.0.0) [36], the linear mixed-models were run with the nlme package (v3.1.164) [37], and the negative binomial mixed-model was run with the lme4 package (v1.1.35.1) [38]. Mixed model tables were generated with the sjPlot package (v2.8.1.5) [39]. Plots related to these models were generated with the ggplot2 package (v3.5.0) [40] and ggthemes package (v5.1.0) [41]. Other data types were analyzed for statistically significant differences using one- or two-way ANOVA or mixed effects analysis with Tukey’s or Dunnett’s multiple comparisons test, or with the non-parametric Wilcoxon’s signed ranks test.

### Weight gain model

For weight gain, we selected a mixed-effect model with fixed effects for sex, age at infection, and the interaction terms between week and treatment. Weight gain was calculated as the percent change compared to baseline weights at 1 week before inoculation. The weight gain model can be written as follows:

$$\begin{gathered} PercentWeight_{i} \sim \alpha_{m[i]} + \delta_{j[i]} + \beta_{1} X_{{Sex_{i} }} + \beta_{2} X_{{AgeAtInfection_{i} }} + \beta_{3} X_{{Week_{i} }} \hfill \\ \quad + \beta_{4} X_{{LRT_{i} }} + \beta_{5} X_{{URT_{i} }} + \beta_{7} X_{Week_{i}} X_{{LRT_{i} }} + \beta_{8} X_{{Week_{i} }} X_{{URT_{i}}} +\epsilon_{i} \hfill \\ \end{gathered}$$

$$\alpha_{m} \sim N\left( {0,\sigma_{ewe}^{2} } \right)$$

$${\updelta }_{j}\sim N\left(0,{\upsigma }_{lambID}^{2}\right)$$

$${\upepsilon }_{i}\sim N\left(0,{\upsigma }^{2}{e}^{2\uptheta *{X}_{AgeAt Infection_{i}}}\right)$$

$$cor\left({\upepsilon }_{t_1},{\upepsilon }_{t_2}\right)=\left\{\begin{array}{c}1\space if \space t_1 =t_2\\ {\uprho }^{\left|t_2-t_1\right|} otherwise\end{array}\right.$$

where *i* represents the individual observation, *m* represents the ewe, *j* represents the lamb ID, *t* represents the time in weeks, and *N* represents a normal distribution.

### Health score model

In the total health score statistical modeling analysis, we used a generalized linear mixed-model with a negative binomial distribution with fixed effects for sex, age at infection, and the interaction terms between week post-inoculation (pi) and treatment. The negative binomial distribution was used because the variance was determined to be much larger than the mean, suggesting a negative binomial distribution rather than a Poisson distribution.

The health score model can be written as follows:

$$\begin{gathered} \log \left( {\mu_{i} } \right) \sim \alpha_{m[i]} + \delta_{j[i]} + \beta_{1} X_{{Sex_{i} }} + \beta_{2} X_{{AgeAtInfection_{i} }} + \beta_{3} X_{{Week_{i} }} \hfill \\ \quad + \beta_{4} X_{{LRT_{i} }} + \beta_{5} X_{{URT_{i} }} + \beta_{6} X_{Week_{i}} X_{{LRT_{i} }} + \beta_{7} X_{{Week_{i} }} X_{{URT_{i} }} \hfill \\ \end{gathered}$$

$${\upalpha }_{m} \sim N\left( {0,{\upsigma }_{ewe}^{2} } \right)$$

$${\updelta }_{j}\sim N\left(0,{\upsigma }_{lambID}^{2}\right)$$

$$TotalScor{e}_{i}\sim NB\left({\upmu }_{i},\upphi \right)$$

where *i* represents the observation, *N* represents a normal distribution, and *NB* represents a negative binomial distribution. The overdispersion parameter $\upphi$ that relates the level of overdispersion in our negative binomial model to the Poisson distribution is estimated to be 0.90.

### Body temperature model

For body temperature post inoculation, we chose a mixed-effect model with fixed effects for sex, age at infection and the interaction terms between week and treatment. The body temperature model can be written as follows:

$$\begin{gathered} Temperature_{i} \sim \alpha_{m[i]} + \delta_{j[i]} + \beta_{1} X_{{Sex_{i} }} + \beta_{2} X_{{AgeAtInfection_{i} }} + \beta_{3} X_{{Week_{i} }} \hfill \\ \quad + \beta_{4} X_{{LRT_{i} }} + \beta_{5} X_{{URT_{i} }} + \beta_{7} X_{Week_{i}} X_{{LRT_{i} }} + \beta_{8} X_{{Week_{i} }} X_{{URT_{i} }} + \epsilon_{i} \hfill \\ \end{gathered}$$

$${\alpha }_{m}\sim N\left(0,{\sigma }_{ewe}^{2}\right)$$

$${\delta }_{j}\sim N\left(0,{\sigma }_{lambID}^{2}\right)$$

$${\epsilon }_{i}\sim N\left(0,{\sigma }^{2}\right)$$

where *i* represents the observation, *m* represents the ewe, *j* represents the lamb ID, and *N* represents a normal distribution.

## Results

### Increased pathogen load in the lower respiratory tract following lower-respiratory tract inoculation of lambs with *Mycoplasma ovipneumoniae*

**Figure 1:**
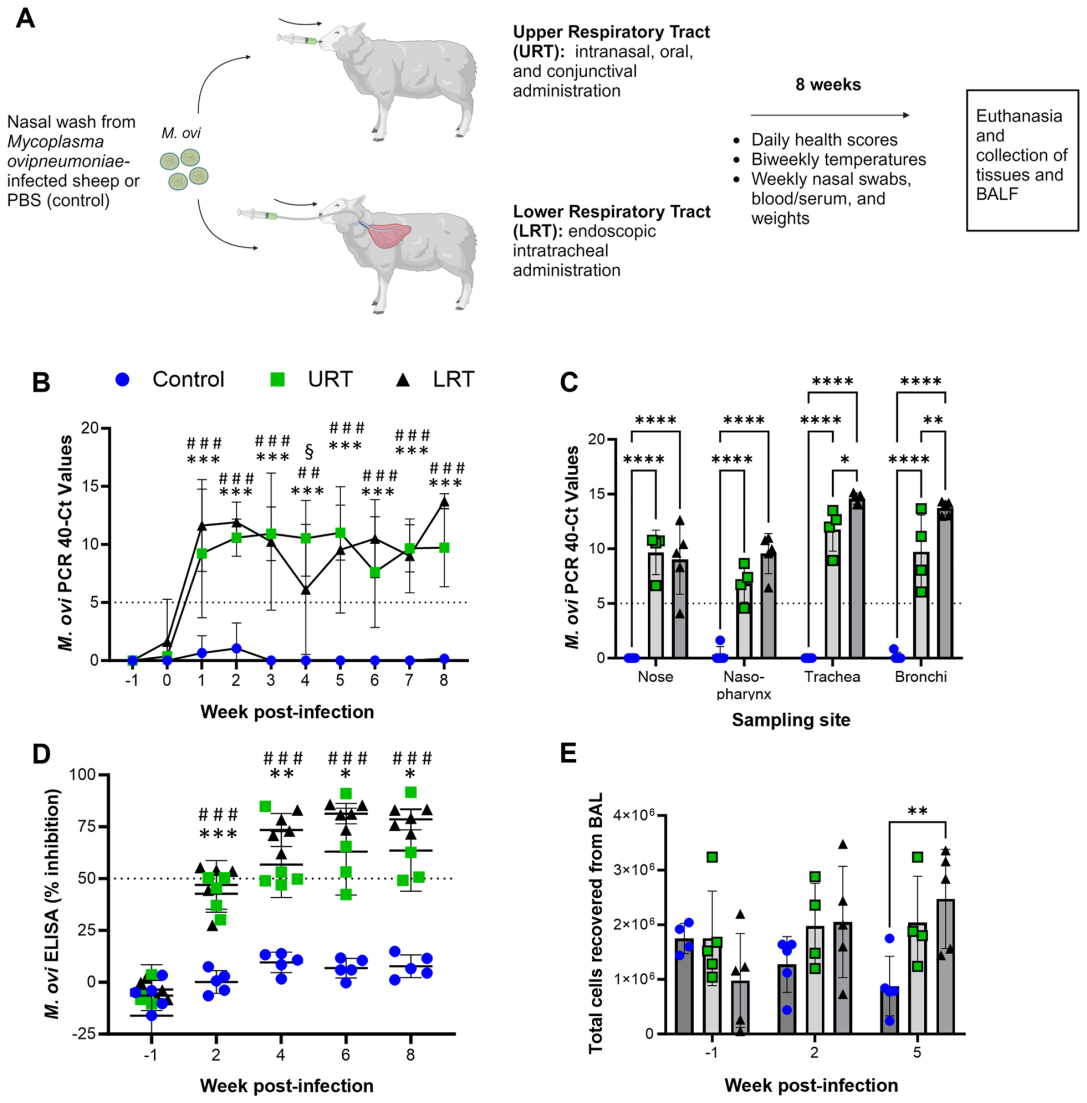
**Both upper and lower respiratory tract pathogen delivery leads to stable colonization with *M. ovipneumoniae*.** **A** Experimental design. Five three-month-old lambs per group were inoculated with PBS or with Ceftiofur-treated nasal wash fluid collected from sheep with natural *M. ovipneumoniae* infection via the upper (URT) or the lower respiratory tract (LRT) and then monitored over eight weeks. Created with BioRender.com. **B** *M. ovipneumoniae* infection levels in nasal swab samples were determined by qPCR. Data are shown as 40 minus Ct value. Mean ± SD. Statistically significant differences between control and URT (*** *P* ≤ 0.001), control and LRT (^###^ *P* ≤ 0.001), and LRT and URT (^§^ *P* ≤ 0.05) were determined by mixed-effects analysis with Tukey’s multiple comparisons test. **C** *M. ovipneumoniae* infection levels in swab samples collected at necropsy from the nose, nasopharynx, trachea, and bronchi were determined by qPCR. Individual data points, mean ± SD. Statistically significant differences (**** *P* ≤ 0.0001, ** *P* ≤ 0.01, * *P* ≤ 0.05) were determined by 2-way ANOVA with Tukey’s multiple comparisons test. **D** *M. ovipneumoniae*-specific antibody levels were determined by competitive ELISA analysis of serum samples before (week-1) and after URT or LRT inoculation with *M. ovipneumoniae* or PBS (control). Statistically significant differences between control and URT (* *P* ≤ 0.05, ** *P* ≤ 0.01, *** *P* ≤ 0.001), or control and LRT (^###^ *P* ≤ 0.001) were determined by mixed-effects analysis with Tukey’s multiple comparisons test. Individual data points, mean ± SD. **E** Total cell counts in bronchoalveolar lavage fluid (BAL) collected via endoscope one week before and two or five weeks after experimental inoculation of lambs. Individual data points, mean ± SD. Statistically significant differences between control and LRT (^##^ *P* ≤ 0.01) were determined by 2-way ANOVA with Tukey’s multiple comparisons test.

In a previous study, we showed that experimental *M. ovipneumoniae* infection, achieved by inoculating the nasal, oral and conjunctival mucosae of specific pathogen-free lambs with ceftiofur-treated nasal wash fluid from conventional lambs with natural *M. ovipneumoniae* infection, caused chronic colonization of the nasal cavity in the absence of clinical signs [21]. Here, we compared inoculation of the nasal, oral, and conjunctival mucosae, i.e., the upper respiratory tract (URT), to endoscopic intratracheal inoculation of the lower respiratory tract (LRT; Figure 1A). Analysis of nasal swab samples for *M. ovipneumoniae* showed that both URT and LRT delivery of *M. ovipneumoniae*-positive, antibiotic-treated nasal wash fluid resulted in consistent colonization of the nasal mucosae with *M. ovipneumoniae* over eight weeks (Figure 1B). Interestingly, swab samples collected at necropsy from both URT (nose and nasopharynx) and LRT (bronchi, and trachea) at eight weeks pi were positive for *M. ovipneumoniae* in lambs from both infection groups, suggesting that inoculation of the URT also leads to infection of the LRT, and vice versa (Figure 1C). However, *M. ovipneumoniae* pathogen load in the bronchi and trachea, i.e., the LRT, was significantly higher in the LRT than the URT group, confirming that delivery route influenced pathogen distribution. None of the samples from control lambs that had received PBS tested *M. ovipneumoniae*-positive at any time during the study. We also analyzed sera collected throughout the study for antibodies to *M. ovipneumoniae* to elucidate whether pathogen delivery route affected the induction of humoral immunity*.* Both URT and the LRT inoculation with *M. ovipneumoniae* led to significant induction of anti-*M. ovipneumoniae* antibodies within two weeks that plateaued by six weeks pi and remained high at eight weeks pi (Figure 1D). There was a non-significant trend for higher antibody responses in the LRT compared to the URT groups. Bronchoalveolar lavage fluids (BALF) were collected at one week before and at two and five weeks pi, and total cell counts were determined. No significant differences in total cell counts were found between experimental groups at −1 and + 2 weeks. However, at five weeks post infection, BALF cell counts were significantly higher in the LRT group (2.47 ± 0.91 × 10^6^) compared to the control group (0.88 ± 0.55 × 10^6^, *P* = 0.0073), with intermediate cell numbers recovered in the URT group (2.04 ± 0.85 × 10^6^; Figure 1E). These data indicate that pathogen delivery route alters pathogen distribution across the respiratory tract and that LRT delivery of *M. ovipneumoniae* induces an increased local response.

### Lower respiratory tract inoculation of lambs with *M. ovipneumoniae* leads to reduced weight gain and more severe clinical signs

**Figure 2:**
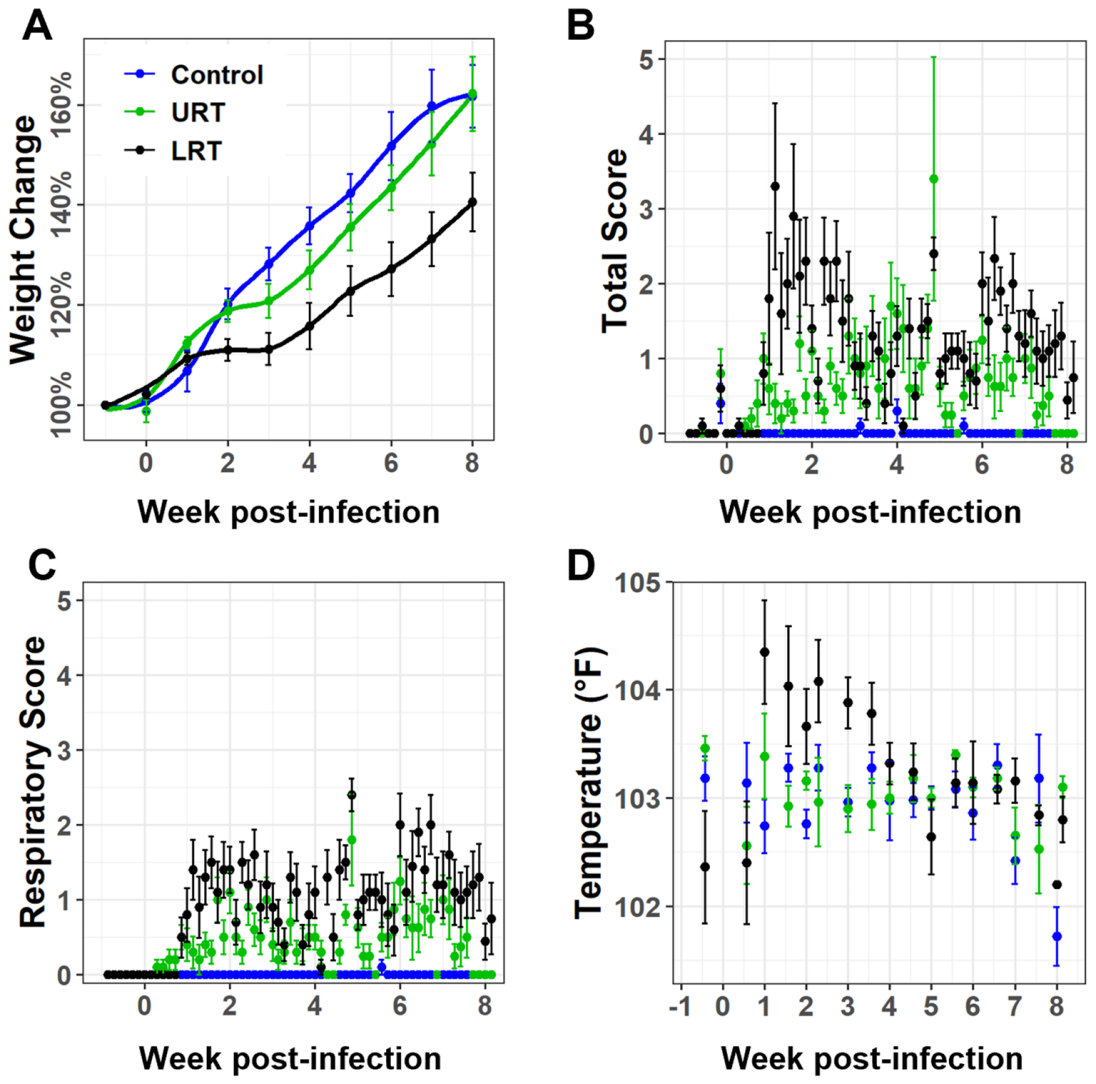
**Lower respiratory tract inoculation with *M. ovipneumoniae* leads to increased respiratory disease and decreased weight gains compared to upper respiratory tract inoculation**. **A** Development of lamb body weights over time, shown as percent change from baseline weight measured one week before inoculation with *M. ovipneumoniae* or PBS. Graph shows predicted mean (line) and SD. **B**, **C** All lambs were screened twice daily for signs of respiratory disease, for changes in behavior and appetite, and administered medications were also recorded (see Additional file 1 for scoring rubric). Daily scores were the sum of the two individual measurements. **B** Total health scores, calculated as the sums of the daily scores in the four categories. **C** Respiratory scores. **D** Biweekly rectal temperatures measured in the three different treatment groups. Graphs show mean ± SD of five lambs per group.

To assess the impact of *M. ovipneumoniae* infection on growth and productivity, we measured body weights of the experimental animals weekly over the course of the study. Interestingly, lambs in the LRT group gained less weight by week than lambs in the control group, while the URT group had intermediate weight gains (Figure 2A). In support of these observations, our weight gain model showed strong evidence for an interaction between week and LRT treatment that corresponded to a 3% decrease in weight gain per week in LRT lambs compared to the control animals (Wald’s test, *P* < 0.001) after accounting for sex and age at infection (Table 4, Additional file 2). Interestingly we also observed strong evidence to suggest that older lambs had lower weight gains, with a one day higher age at infection associated with a 1% decrease in percent weight gain (Wald’s test, *P* < 0.01).

**Table 4:** **Weight gain model**

Predictors	Estimates	CI	*P*
(Intercept)	1.42	1.17 to 1.67	** < 0.001**
Sex [m]	0.03	−0.38 to 0.44	0.538
Age at infection	−0.01	−0.01 to −0.00	**0.009**
Week	0.07	0.06 to 0.08	**< 0.001**
Treatment [URT]	−0.00	−0.58 to 0.58	0.998
Treatment [LRT]	−0.07	−0.72 to 0.58	0.398
Treatment [URT] x Week	0.00	−0.01 to 0.02	0.702
Treatment [LRT] x Week	−0.03	−0.04 to −0.01	** < 0.001**
N_Ewe_	11		
N_lambID_	15		
Observations	130		

Mixed linear model coefficients for weight development based on percent weight change. *P* values considered statistically significant are bolded.

We also monitored lamb health, behavior and food intake two times per day, using our previously published scoring system [21] with slight modifications (Additional file 1). In contrast to our earlier study [21], all lambs that had received the *M. ovipneumoniae-*containing nasal wash fluid developed significant respiratory disease (Figures 2B and C). Total clinical scores in the LRT group peaked at two weeks post infection, with a second peak seen at about 7 weeks pi (Figure 2B). In the URT group, clinical scores peaked at 4 and 7 weeks pi (Figure 2B). In particular, respiratory scores, representing incidences of coughing, nasal discharge and/or labored breathing, were significantly higher for infected lambs compared to uninfected controls, again with highest scores seen following LRT inoculation, but with a peak at six weeks post infection (Figure 2C). Conversely, no apparent changes in behavior, appetite, or administered medications were detected, although the control lambs consistently scored lower than the infected lambs (Additional file 3). Consistent with these observations, our health score model found a 29.2-times increase in the total score between the control group and the LRT group (Wald’s test, *P* < 0.001) and a 15.2-times increase in total score between the control group and the URT group (Wald’s test, *P* < 0.001) (Table 5, Additional file 2). This corresponded to a 38% increase in total health scores per week for the URT and LRT groups (Wald’s test, *P* = 0.03; Additional file 2, Table 5).

**Table 5:** **Health score model**

Predictors	Incidence rate ratios	CI	*P*
(Intercept)	0.02	0.00–0.23	**0.001**
Sex [m]	1.41	0.88–2.27	0.157
Age at infection	1.00	0.97–1.03	0.828
Treatment [URT]	15.16	5.12–44.86	** < 0.001**
Treatment [LRT]	29.18	10.00–85.16	** < 0.001**
Week	0.81	0.61–1.08	0.148
Treatment [URT] x Week	1.38	1.03–1.85	**0.029**
Treatment [LRT] x Week	1.38	1.04–1.85	**0.028**
Observations	1840		

Mixed linear model coefficients for health scores. *P* values considered statistically significant are bolded.

Lambs in the LRT group also had increased incidences of elevated body temperatures that exceeded 104 °F (40 °C) at multiple time points, whereas body temperatures remained in the normal range in the URT and control groups (Figure 2D). However, our body temperature model (Table 6), showed now significant difference in temperature between the control animals and the URT or LRT treatments (Wald’s test, *P* > 0.05). In general, lamb body temperatures decreased slightly over time, with moderate evidence for a 0.07 °C decrease (Wald’s test, *P* = 0.038) after accounting for treatment, age at infection, and sex. In summary, data analysis using mixed linear modeling confirmed that the site of pathogen introduction to the respiratory tract can significantly impact disease severity in *M. ovipneumoniae-*infected lambs.

**Table 6:** **Body temperature model**

Predictors	Estimates	CI	*P*
(Intercept)	102.78	101.43–104.12	** < 0.001**
Sex [m]	−0.13	−2.04–1.77	0.540
Age at infection	0.01	−0.02–0.03	0.457
Treatment [URT]	−0.05	−3.08–2.98	0.865
Treatment [LRT]	0.69	−2.29–3.68	0.208
Week	−0.07	−0.13 to −0.00	**0.038**
Treatment [URT] x Week	0.03	−0.06–0.12	0.529
Treatment [LRT] x Week	−0.07	−0.16–0.02	0.148
N _Ewe_	11		
N _lambID_	15		
Observations	241		

Mixed linear model coefficients for body temperatures. *P* values considered statistically significant are bolded.

### Lower respiratory tract inoculation of lambs with *M. ovipneumoniae* was associated with gross and microscopic lung pathology

**Figure 3:**
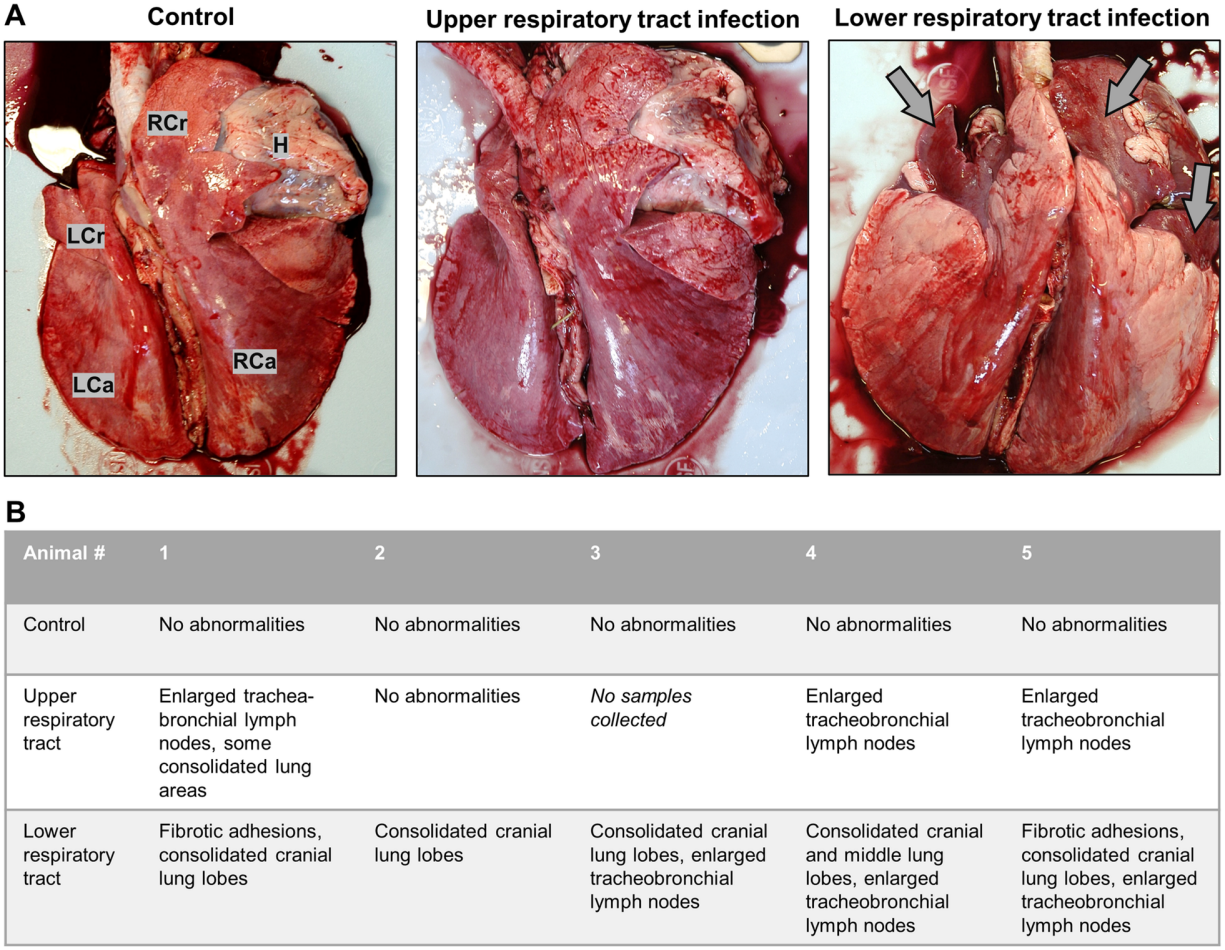
**Increased gross pathological changes in cranial lung lobes of lambs after lower respiratory tract, but not upper respiratory tract inoculation with *M. ovipneumoniae****.* **A** Representative lungs from lambs with upper and lower respiratory tract inoculation at eight weeks post inoculation with PBS or with *M. ovipneumoniae*. LCr—left cranial lung, RCr—right cranial lung, LCa—left caudal lung, RCa—right caudal lung, H heart. Arrows in the right panel point to consolidated lung tissue. **B** Gross pathological findings for lungs from individual animals at necropsy. One animal in the URT group developed a urinary tract disease and was euthanized prior to the experimental endpoint, so that no samples could be collected.

Lungs collected after euthanasia at 8 weeks pi were analyzed for gross and microscopic abnormalities. The respiratory tract of the control lambs had no visible abnormalities. In contrast, enlarged tracheobronchial lymph nodes were found in three out of four lambs in the URT inoculation group, and patchy areas of consolidated lung tissue were present in one lamb (Figures 3A and B). More strikingly, all lambs in the LRT inoculation group (5/5) had consolidated cranial lung lobes, three had enlarged tracheobronchial nodes, and two had fibrotic lung adhesions, all indicative of significant pneumonia (Figures 3A and B).

**Figure 4:**
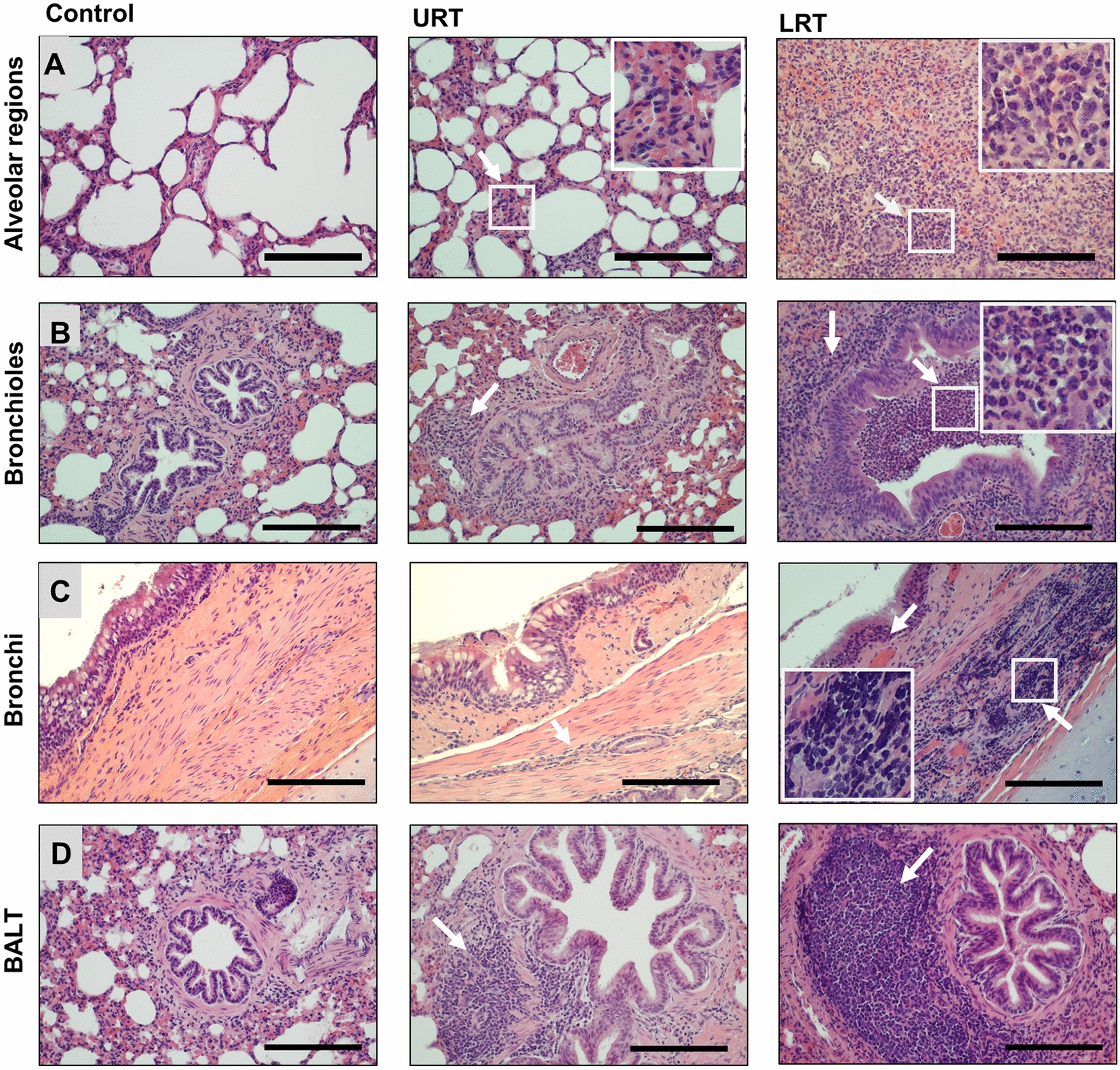
**Increased histopathological changes in cranial lung lobes of lambs after lower respiratory tract compared to upper respiratory tract inoculation with** ***M. ovipneumoniae***. Histopathological analysis of paraffin-embedded, H&E-stained sections of respiratory tract tissues collected upon necropsy at eight weeks post inoculation. **A** Representative images of the alveolar regions with interstitium show healthy tissue from a representative control lamb. Samples from *M. ovipneumoniae-*infected animals show thickening of the alveolar septa and infiltration of alveolar spaces with neutrophils and mononuclear cells that were mild to moderate in the URT group and severe in the LRT group. **B** Bronchioles show peribronchiolar lymphoid cuffs in the URT and LRT groups and severe luminal infiltration with neutrophils and necrotic material in the LRT group. (**C**) Severe submucosal inflammatory infiltrates in the bronchial submucosa of a lamb from the LRT group. Some infiltrating immune cells are also present in tissue from the URT group. (**D**) Formation of bronchus-associated lymphoid tissue (BALT) is observed in the URT and LRT groups, but not in the control group. All bars are 100 µm.

These observations were confirmed by histopathological analysis of lung tissue and bronchial regions (Figure 4 and Table 7). A significant increase in interstitial, alveolar, and bronchiolar inflammation was detected in the cranial lungs of the LRT group compared to the control group. Cranial lungs of the URT group also had significantly increased alveolar and interstitial inflammation compared to the control group, with a trend for increased bronchiolar inflammation (Figures 4A and B). Some bronchioles in lambs from the LRT infection group were filled with large numbers of neutrophilic granulocytes, indicative of significant acute inflammation (Figure 4B, Table 7). We also observed inflammatory infiltrates around the bronchi of *M. ovipneumoniae*-infected lambs that were mild in the URT group, moderate to severe in the LRT group, but absent from healthy or control lambs (Figure 4C). Interestingly, bronchus-associated lymphoid tissue (BALT) developed to a similar extent in both the URT and LRT groups but was generally absent from lungs of control animals (Figure 4D, Table 7). No significant differences were found for any group or parameter for the caudal lung lobes, indicating that the disease was limited to the cranial lung (data not shown). Overall, these data indicate that the increased pathogen load in the lower respiratory tract of lambs in the LRT inoculation group was associated with more severe respiratory pathology, but that BALT was induced independent of inoculation route or pathogen load.

**Table 7:** **Histopathological scoring of lungs from sheep infected with PBS (control) or with** ***M. ovipneumoniae-* containing nasal-wash fluids through inoculation of the upper (URT) or lower respiratory tract (LRT)**

	Control (*n* = 5)	URT (*n* = 4)	LRT (*n* = 5)	*P-*value*
Pulmonary edema and congestion				
*Cranial*	3.4 ± 1.1	3.8 ± 0.5	3.2 ± 0.8	0.6631
* Caudal*	3.2 ± 0.8	3.5 ± 0.6	3.6 ± 0.9	0.7212
Alveolar and interstitial inflammation				
*Cranial*	0.8 ± 1.1^a^	2.8 ± 0.5^b^	5.0 ± 1.2^c^	**0.0002**
* Caudal*	0.2 ± 0.4	0.8 ± 1.0	1.0 ± 0.0	0.1207
Bronchiolar inflammation				
* Cranial*	0.2 ± 0.4^a^	1.5 ± 0.6^a^	4.4 ± 1.5^b^	**0.0001**
* Caudal*	0.0 ± 0.0	0.0 ± 0.0	0.2 ± 0.4	0.4406
BALT hyperplasia				
* Cranial*	0.6 ± 0.5^a^	3.3 ± 1.0^b^	4.2 ± 0.8^b^	** < 0.0001**
* Caudal*	0.4 ± 0.5	1.0 ± 0.0	1.2 ± 0.8	0.1406
Total score	8.8 ± 3.1^a^	11.3 ± 1.3^a^	22.8 ± 4.1^b^	** < 0.0001**

^*^ANOVA analysis. *P* values considered statistically significant are bolded.

^a,b,c^Different letters denote statistically significant differences (*P* < 0.05) in Tukey’s pairwise comparisons test.

### *M. ovipneumoniae* infection induced significantly increased IFN-γ gene expression in the blood and lungs

**Figure 5:**
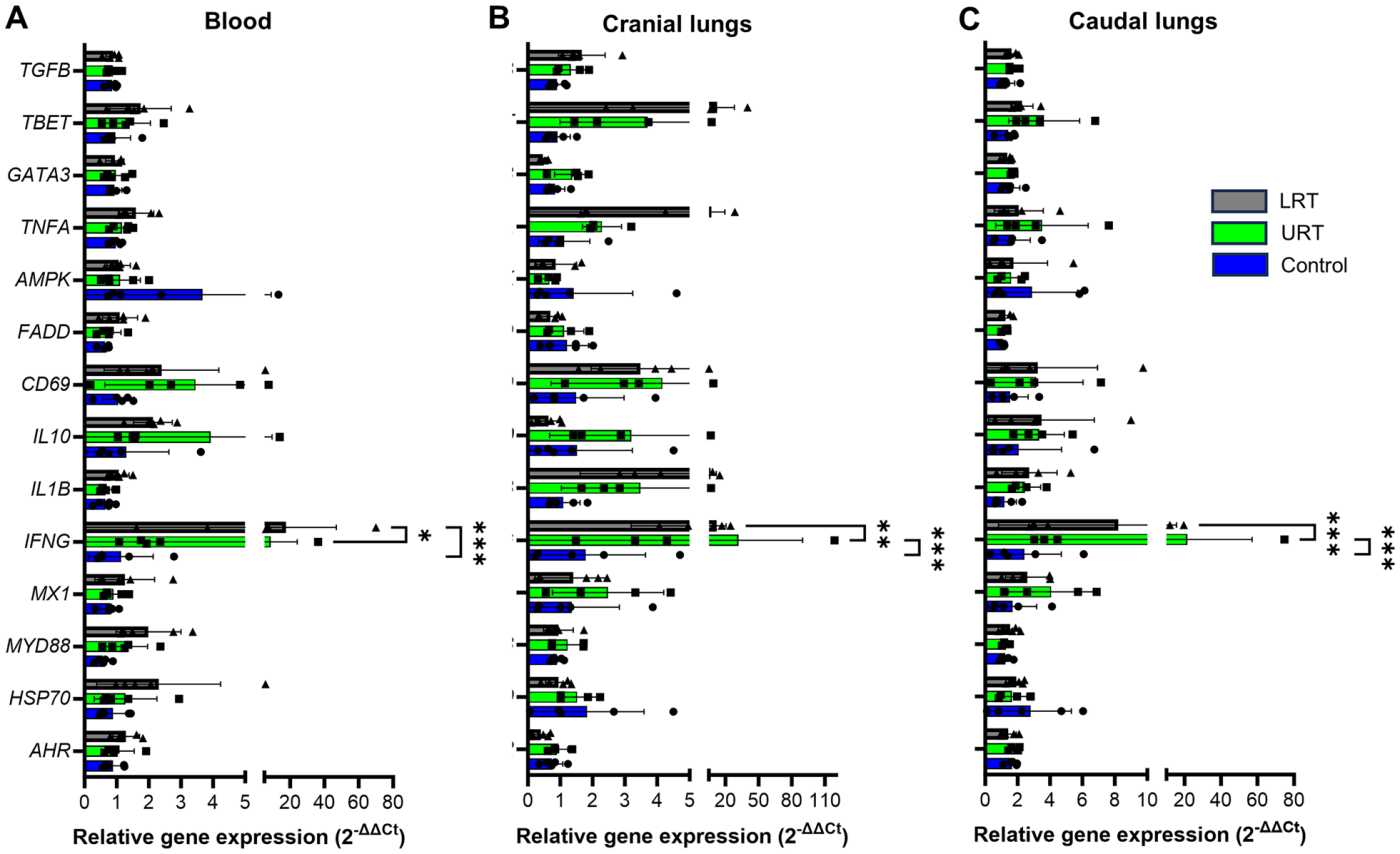
***M. ovipneumoniae* infection drives IFN-γ gene expression in blood and lungs.** Gene expression analysis for a panel of immune related genes [34] was performed using quantitative RT-PCR, with the delta-delta Ct method for data analysis. **A** Whole blood samples were collected at weeks 0, 2, 4, 6 and 8 pi. Datapoints for each lamb represent average expression for weeks 2, 4, 6 and 8 pi normalized to the baseline samples collected at week zero before inoculation. **B**, **C** RNA was isolated from cranial and caudal lung tissues collected at necropsy (week 8 pi). Data were normalized to the average gene expression of the control group. For all three panels, individual data points, mean and SD are shown. Data were analyzed by 2-way ANOVA with Tukey’s multiple comparisons test (* *P* ≤ 0.05, ** *P* ≤ 0.01, *** *P* ≤ 0.001).

A transcriptomics panel for fourteen immune related genes was applied to tissue samples from cranial and caudal lungs at necropsy (8 weeks pi) and to blood samples collected at 0, 2, 4, 6, and 8 weeks pi [34]. Interestingly, *M. ovipneumoniae* infection was associated with increased expression of *IFNG* in all three types of samples (Figure 5). For blood samples, no clear trends over time were apparent, so that gene expression from the 2, 4, 6, and 8 week time points relative to baseline expression at week 0 was averaged for each animal (Figure 5A). Blood samples from LRT lambs showed a significant increase in IFN-γ gene expression compared to the URT group (*P* ≤ 0.05) and compared to the control group (*P* ≤ 0.001). In addition, there was a trend for increased expression of the T cell activation marker *CD69* in blood from both URT and LRT lambs compared to the control group, and a trend for increased expression of the anti-inflammatory cytokine *IL10* in lambs from the URT group alone that was also seen in cranial lung tissue. Interestingly, both cranial and caudal lung tissue from lambs inoculated via the URT showed significantly increased expression of *IFNG* compared to the control group (Figures 5B and C). *IFNG* expression also was increased in the LRT group, but this was not significant. No other significant changes were detected. Both cranial and caudal lungs from URT and LRT lambs showed a trend for increased expression of *CD69.* Cranial lung tissue from URT and LRT lambs also showed a trend for increased expression of the Th1 transcription factor *TBET* and the pro-inflammatory cytokines *TNFA* and *IL1B.* Overall, these data suggest that *M. ovipneumoniae* infection may enhance type 1 immunity both locally and systemically [42].

### Inoculation of specific pathogen free domestic lambs with ceftiofur-treated nasal wash fluid resulted in co-infection with *M. ovipneumoniae *and *Pasteurellacea*

Given that our previous studies investigating monoinfection with *M. ovipneumoniae* resulted in subclinical colonization [21, 30], whereas the lambs exhibited significant clinical signs in the current experiment, we asked whether additional respiratory pathogens were present that might have contributed to disease pathogenesis. The SPF flock used in this study consistently tested negative for nasal *Pasteurellacea* and *M. ovipneumoniae* colonization. Nasal wash fluids used for inoculation had been treated with ceftiofur, which was expected to eliminate respiratory bacterial pathogens other than *Mycoplasma spp.* [12, 43]. However, we found that nasal swabs from all lambs from both the URT and LRT groups, but not the control group, contained culturable *Bordetella sp.* (*bronchiseptica* and/or *parapertussis*) at three weeks pi, with inconsistent detection thereafter (Additional files 4A-C). In addition, *M. haemolytica* was isolated from one lamb in the URT group and three lambs in the LRT group at various time points pi*.* Three isolates of *M. haemolytica* were cultured, and the presence of the leukotoxin gene *lktA* was confirmed by PCR [33] in all isolates (data not shown).

**Figure 6:**
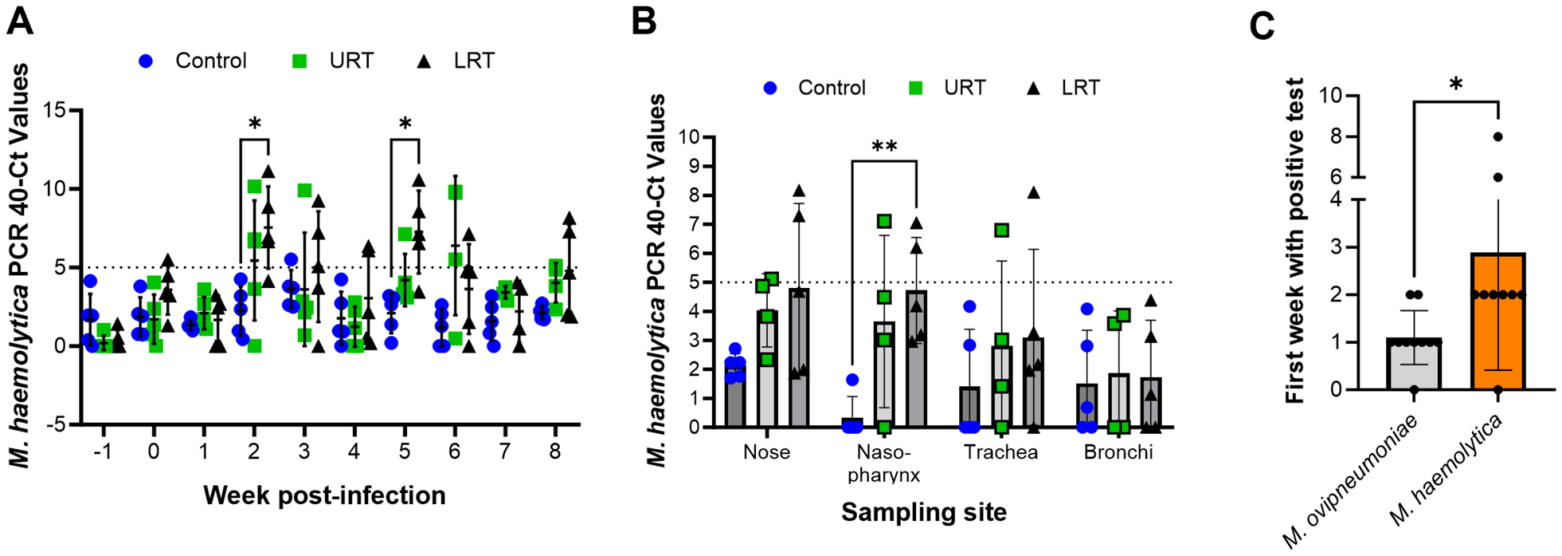
**Identification of *Mannheimia haemolytica* in *M. ovipneumoniae* infected lambs.** **A** *Mannheimia haemolytica* infection levels in nasal swab samples collected throughout the experiment were determined by qPCR. Data are shown as 40 minus Ct; individual data points, mean ± SD. Statistically significant differences between groups (* *P* ≤ 0.05) were determined by mixed-effects analysis with Tukey’s multiple comparisons test. Dotted line indicates limit of detection. **B** *M. haemolytica* infection levels in swab samples collected at necropsy from the nose, nasopharynx, trachea, and bronchi were determined by qPCR. Individual data points, mean ± SD. Statistically significant differences (** *P* ≤ 0.01) were determined by 2-way ANOVA with Tukey’s multiple comparisons test. **C** Timepoint for first detection of a PCR-positive sample (Ct ≤ 35) for *M. ovipneumoniae* or *M. haemolytica* in each animal. Only lambs from the URT and LRT groups were included in the analysis. Individual datapoints, mean ± SD; * *P* ≤ 0.05 Wilcoxon matched-pairs signed rank test.

To further investigate the apparent *M. haemolytica* co-infection in the lambs, we also performed quantitative PCR screening of the nasal swab samples collected throughout the experiment for *M. haemolytica* (Figure 6A). A subset of samples from both groups of inoculated lambs, but not the control group, tested positive for *M. haemolytica* throughout the study*,* with a significant increase in positive samples detected for the LRT group at weeks two and five pi. In general, more *M. haemolytica-*positive samples were detected in the LRT compared to the URT groups (Additional file 4D). Conversely, only a small proportion of swabs collected at the experimental endpoint from the nose, deep nasopharynx, and trachea from URT and LRT lambs tested positive for *M. haemolytica* (Figure 6B), with statistical significance only found for nasopharyngeal swabs collected from LRT group lambs. Surprisingly, we were unable to detect *M. haemolytica* in any swab samples collected from bronchi, even after LRT inoculation.

When looking at associations between *M. ovipneumoniae* and *M. haemolytica* infection, we found that nasal swab samples tested positive for *M. ovipneumoniae* significantly earlier (at 1.1 ± 2.6 weeks pi) than for *M. haemolytica* (at 2.9 ± 2.5 weeks pi; Figure 6C). Across all time points, there was only a weak correlation between *M. ovipneumoniae* and *M. haemolytica* pathogen load based on cT values obtained by qPCR analysis (Additional file 4E).

### Statistical diagnostics assessment

Model diagnostics for each of the mixed-models were assessed, and no severe violations of linearity or normality of residuals were observed for the health score and percent weight models. The temperature model had severe violations of normality, but mixed-models are robust to violations of normality with the resulting effect of these violations being a larger estimate of uncertainty [44]. Multicollinearity was observed in both the body temperature model and the weight gain model, which increases the likelihood of type II error due to a larger confidence interval for the estimates [45].

## Discussion

*Mycoplasma ovipneumoniae* is a facultative pathogen associated with ovine respiratory disease that ranges from asymptomatic colonization to lethal pneumonia [3, 21, 43]. The factors that determine the outcome of infection are still incompletely understood but include bacterial strain characteristics [3], genetic susceptibility of the host [19, 46], and presence of co-infections [15]. In two recent challenge studies that we performed with specific pathogen-free lambs with *M. ovipneumoniae*, the infection had no measurable impact on weights, lamb health, or lung inflammation [21, 30]. In contrast, studies from China described coughing, wheezing, and lethargy in response to infection with the *M. ovipneumoniae* type strain Y-98 [9, 10]. Notably, *M. ovipneumoniae* was directly administered into the bronchi or trachea in these experiments [9, 10], whereas we had used an upper respiratory tract inoculation protocol based on a publication by Besser et al. [12, 21, 30]. Here, we performed a side-by-side comparison of endoscopic LRT inoculation with *M. ovipneumoniae* and inoculation of the URT i.e., the nasal cavity, conjunctiva, and oral cavity.

Under normal circumstances, *M. ovipneumoniae* transmission between sheep is thought to occur via the URT through droplets [3]. However, it is conceivable that *M. ovipneumoniae* may reach the lower airways if the animal is panting due to stress or exertion, if pathogen loads are extremely high, or if mucociliary defense mechanisms are compromised due to ongoing disease processes or infections. PCR analysis of swab samples collected at necropsy confirmed that, compared to URT inoculation, pathogen administration to the LRT via endoscopic intratracheal inoculation indeed led to significantly higher *M. ovipneumoniae* loads in the trachea and bronchi, but similar loads in the nasal cavity and nasopharynx. These results corroborated that different distribution patterns of the pathogen could be effectively modeled using different experimental inoculation methods. Importantly, LRT *M. ovipneumoniae* administration led to significantly more severe clinical disease and increased lung pathology than URT administration. Our results also are consistent with findings from other experimental models that showed that outcomes of respiratory infections can vary depending on where *Mycoplasma* or other pathogens are delivered. In a hamster model of *M. pneumoniae* infection, small particle aerosols and large volume inocula reached the lungs and subsequently caused lung pathology, whereas *Mycoplasma* from small inocula or large particle aerosols remained in the URT and did not cause lung lesions [47]. Likewise, pneumonia in mice inoculated with *Bordetella pertussis* was more severe and less variable following aerosol inoculation compared to intranasal delivery [48]. A study that compared intranasal and intratracheal inoculation of Cebus monkeys with influenza A virus found that only intratracheal pathogen delivery resulted in clinical disease [49]. Together, these findings indicate that circumventing critical protective mechanisms of the upper respiratory tract, such as the tonsils and mucociliary clearance, consistently results in pathogen growth in the lower airways.

Previous reports have shown that clinical *M. ovipneumoniae* infection in both domestic and wild sheep predominantly occurs when the animals are co-infected with *Pasteurellaceae* such as *Mannheimia haemolytica* and *Bibersteinia trehalosi* [15, 17, 50]. Despite treatment of the nasal wash inocula with ceftiofur, we found that all lambs in the *M. ovipneumoniae-*infected groups except one also were infected with leukotoxin-positive *M. haemolytica* and *Bordetella spp.* Both *M. ovipneumoniae* and *Pasteurellaceae* are considered facultative respiratory pathogens but also express virulence factors that can facilitate the expansion of other pathogens: *M. haemolytica* produces a leukotoxin (*lktA*) that targets the integrin CD18 (integrin β2) on leukocytes and causes leukocyte death and local immunosuppression [51, 52], and *M. ovipneumoniae* can produce hydrogen peroxide, which severely disrupts ciliary activity on the respiratory epithelium [28, 53]. The ability of *M. ovipneumoniae* to support expansion of *M. haemolytica* in our study was extremely impressive, since no detectable *M. haemolytica* were present in the original inocula or in any of our SPF sheep prior to the study, and none of the uninfected control lambs ever tested positive for this organism. *M. ovipneumoniae* was generally detected in the nasal swabs at earlier timepoints than the *M. haemolytica*, suggesting that *M. ovipneumoniae* was the driving force for respiratory disease development in our study.

Whether the *M. haemolytica* co-infection contributed to the clinical signs and respiratory pathology observed in the present study remains unclear. Notably, the *M. haemolytica* strains that we recovered from the experimental animals were positive for *lktA*. However, as none of the postmortem swabs obtained from the lower respiratory tract tested positive for *M. haemolytica*, the pulmonary lesions found in our study were likely caused by the *M. ovipneumoniae.* Previous studies have reported conflicting results regarding lung infection with *M. haemolytica* in *M. ovipneumoniae-*infected sheep [11, 33, 54], which suggests that additional factors may determine the relative contributions of *M. ovipneumoniae* and *M. haemolytica* to ovine pneumonia during co-infection. While disease severity in the present infection study was higher than in a previous study performed by our team, where *M. haemolytica* was not detected [21], this difference may have been due to variations in *M. ovipneumoniae* strain virulence, infectious dose, or the lower age of the lambs.

One other key finding from the present study was the observation that *M. ovipneumoniae* infection was associated with increased gene expression of *IFNG* in the blood, the cranial and the caudal lungs. This was significant for the LRT group for blood samples and the URT for lung samples, but similar trends were seen for both infection groups. So far, very little information is available regarding the immune response to *M. ovipneumoniae*. The increase in IFN-γ, which is typically produced by Th1 cells, innate lymphoid cells (ILC) 1, natural killer cells, and cytotoxic T cells, is indicative of a enhanced type 1 immunity, the characteristic response to intracellular pathogens and viruses [42]. While it still remains unclear whether *M. ovipneumoniae* can survive intracellularly, many *Mycoplasma* species including *M. bovis* are known to replicate inside host cells [55, 56], where type 1 immune responses are expected to be protective. In humans, serum IFN-γ is a diagnostically relevant biomarker for pediatric mycoplasma pneumonia, where it significantly correlates with disease severity [57]. A few previous studies have analyzed cytokine responses in *M. ovipneumoniae*-infected sheep, with highly variable results. Bowen et al. [34] analyzed blood samples from adult Bighorn sheep with acute or chronic *M. ovipneumoniae* and found that *MX1, TGFB*, and *IL1B* gene expression increased as infection progressed, while *IFNG* was only increased in early infection. Conversely, bulk RNASeq and pathway analysis of lung tissue analyzed at 4 and 14 days pi revealed that *M. ovipneumoniae* infection predominantly led to an increased expression of toll-like receptor pathway genes in Bashbay sheep and of primary immunodeficiency genes in Argali hybrid sheep [9, 10]. Li et al. [58] analyzed the respiratory tracts of domestic sheep naturally infected with *M. ovipneumoniae* and found increased gene expression of *IFNG* in the trachea, but not the lungs, whereas the expression of *IL1B* and *TNFA* was increased across the entire respiratory tract. These different findings may be related to differences in sheep species and experimental setup between the studies.

Our study did have several limitations. First, although we did find significant differences in pathogen distribution between lambs inoculated via LRT vs. URT, *M. ovipneumoniae* was still detected in samples from all anatomical sites in both groups. Second, although we and others have previously performed successful monoinfection of lambs with *M. ovipneumoniae* using ceftiofur-treated nasal washes from naturally infected animals [12, 21, 43, 59], we achieved an unintentional polymicrobial infection in the present study, as discussed above. Third, the immunological screening only included complex samples, i.e. whole blood and lung, which contain many different cell types, and was limited to gene expression analysis, which does not necessarily correspond to functional cytokine levels in these compartments. Fourth, the statistical models were found to have some violations of normality (body temperature model) or multicollinearity (body temperature and weight gain models), which increases the likelihood of a false negative for both of these models. Last, while we did perform both random selection and random assignment of the animals to the treatment groups, the limited number of animals in each group does decrease the scope of inference for our results.

In summary, our study showed that pathogen delivery route impacts disease severity in experimental infection of sheep with *M. ovipneumoniae.* Specifically, deep intratracheal inoculation with the organisms resulted in more severe clinical disease, reduced weight gains, increased macroscopic and microscopic lung pathology, and increased pathogen load in the LRT compared to inoculation of URT mucosal surfaces. Clinical respiratory disease involved lower weight gains in the lambs, confirming the negative impact of *M. ovipneumoniae* infection on productivity [1, 2].

## Supplementary Information

**Additional file 1: Rubric for health scoring.** Scoring rubric for lamb health during challenge experiment, based on Johnson et al. [21].

**Additional file 2: Linear mixed modeling of lamb weight development and health.** (**A**) The predicted percent weight change effect is shown for the treatments across the weeks while holding sex and age at infection constant. The grey shaded area corresponds to the 95% confidence interval. (**B**) The predicted total score effect is shown for each treatment group across the weeks post-infection holding sex and age at infection constant. The grey shaded area corresponds to the 95% confidence interval. (**C**) The predicted total score effect is shown for just the treatment groups while holding week, sex, and age at infection constant, the lines correspond to the 95% confidence interval.

**Additional file 3: Health scores for behavior, appetite, and administered medications, and body temperatures.** All lambs were screened twice daily for changes in (**A**) behavior, and (**B**) appetite. (**C**) Administered medications were also recorded (see Additional file 1 for scoring rubric). Daily scores were the sum of the two individual measurements. Graphs show mean ± SD of five lambs per group.

**Additional file 4: Identification of Pasteurellacea in *M. ovipneumoniae-*infected lambs.** Routine bacteriological analysis of nasal swab samples collected throughout the study from lambs in (**A**) the control group, (**B**) the upper respiratory tract (URT) infection group, and (**C**) the lower respiratory tract (LRT) infection group for the presence of *Pasteurellacea* was performed at the Washington Animal Diseases Diagnostic Laboratory. (**D**) Percentage of *Mannheimia haemolytica* positive nasal swab samples out of all samples in lambs from the three experimental groups. (**E**) Weak correlation between Ct values from *M. ovipneumoniae* and *M. haemolytica* PCRs across all animals from the URT and LRT groups and all time points post inoculation. Data were analyzed by simple linear regression analysis.

## Data Availability

The datasets used and/or analyzed during the current study are available from the corresponding author on reasonable request.

## References

[1] Besser TE, Levy J, Ackerman M, Nelson D, Manlove K, Potter KA, Busboom J, Benson M (2019) A pilot study of the effects of *Mycoplasma ovipneumoniae* exposure on domestic lamb growth and performance. PLoS One 14:e0207420 30730893 10.1371/journal.pone.0207420 PMC6366759

[2] Manlove K, Branan M, Baker K, Bradway D, Cassirer EF, Marshall KL, Miller RS, Sweeney S, Cross PC, Besser TE (2019) Risk factors and productivity losses associated with *Mycoplasma ovipneumoniae* infection in United States domestic sheep operations. Prev Vet Med 168:30–38 31097121 10.1016/j.prevetmed.2019.04.006

[3] Maksimović Z, Rifatbegović M, Loria GR, Nicholas RAJ (2022) *Mycoplasma ovipneumoniae*: a most variable pathogen. Pathogens 11:1477 36558811 10.3390/pathogens11121477 PMC9781387

[4] USDA (2013) Sheep 2011. Part III: Health and Management Practices on U.S. Sheep Operations, 2011. In*.*: USDA–APHIS–VS–CEAH–NAHMS. Fort Collins, CO

[5] Gaeta NC, de Sa Guimaraes AM, Timenetsky J, Clouser S, Gregory L, Ganda E (2022) The first *Mycoplasma ovipneumoniae* recovered from a sheep with respiratory disease in Brazil - draft genome and genomic analysis. Vet Res Commun 46:1311–1318 35804255 10.1007/s11259-022-09972-x

[6] Waheed Khudiar Khareeb Z, Ismail Ibrahim Z, Ali Abdullah F (2022) Pathological and molecular detection of *Mycoplasma ovipneumoneae* in Sheep, Basrah Province, Iraq. Arch Razi Inst 77:2073–2080 37274899 10.22092/ARI.2022.357996.2134 PMC10237560

[7] Deeney AS, Collins R, Ridley AM (2021) Identification of Mycoplasma species and related organisms from ruminants in England and Wales during 2005–2019. BMC Vet Res 17:325 34641885 10.1186/s12917-021-03037-y PMC8513359

[8] Zhao JY, Du YZ, Song YP, Zhou P, Chu YF, Wu JY (2021) Investigation of the prevalence of *Mycoplasma Ovipneumoniae* in Southern Xinjiang, China. J Vet Res 65:155–160 34250299 10.2478/jvetres-2021-0021 PMC8256467

[9] Li Z, Du Z, Sun Y, Wang J, Liu H, Yang Y, Zhao N (2020) Comprehensive RNA-Seq profiling of the lung transcriptome of Argali hybrid sheep in response to experimental *Mycoplasma ovipneumoniae* infection. Res Vet Sci 132:57–68 32505020 10.1016/j.rvsc.2020.05.014

[10] Du Z, Sun Y, Wang J, Liu H, Yang Y, Zhao N (2020) Comprehensive RNA-Seq profiling of the lung transcriptome of Bashbay sheep in response to experimental *Mycoplasma ovipneumoniae* infection. PLoS One 15:e0214497 32639963 10.1371/journal.pone.0214497 PMC7343132

[11] Buddle BM, Herceg M, Davies DH (1984) Experimental infection of sheep with *Mycoplasma ovipneumoniae* and *Pasteurella haemolytica*. Vet Microbiol 9:543–548 6506448 10.1016/0378-1135(84)90016-6

[12] Besser TE, Cassirer EF, Potter KA, Lahmers K, Oaks JL, Shanthalingam S, Srikumaran S, Foreyt WJ (2014) Epizootic pneumonia of bighorn sheep following experimental exposure to *Mycoplasma ovipneumoniae*. PLoS One 9:e110039 25302992 10.1371/journal.pone.0110039 PMC4193846

[13] Jones GE, Gilmour JS, Rae AG (1982) The effects of different strains of *Mycoplasma ovipneumoniae* on specific pathogen-free and conventionally-reared lambs. J Comp Pathol 92:267–272 6211467 10.1016/0021-9975(82)90085-8

[14] Foggie A, Jones GE, Buxton D (1976) The experimental infection of specific pathogen free lambs with *Mycoplasma ovipneumoniae*. Res Vet Sci 21:28–35 133436

[15] Besser TE, Frances Cassirer E, Highland MA, Wolff P, Justice-Allen A, Mansfield K, Davis MA, Foreyt W (2013) Bighorn sheep pneumonia: sorting out the cause of a polymicrobial disease. Prev Vet Med 108:85–93 23253148 10.1016/j.prevetmed.2012.11.018

[16] Brogden KA, Lehmkuhl HD, Cutlip RC (1998) *Pasteurella haemolytica* complicated respiratory infections in sheep and goats. Vet Res 29:233–254 9689740

[17] Dassanayake RP, Shanthalingam S, Herndon CN, Subramaniam R, Lawrence PK, Bavananthasivam J, Cassirer EF, Haldorson GJ, Foreyt WJ, Rurangirwa FR, Knowles DP, Besser TE, Srikumaran S (2010) *Mycoplasma ovipneumoniae* can predispose bighorn sheep to fatal *Mannheimia haemolytica* pneumonia. Vet Microbiol 145:354–359 20466492 10.1016/j.vetmic.2010.04.011

[18] Johnson BM, Stroud-Settles J, Roug A, Manlove K (2022) Disease ecology of a low-virulence *Mycoplasma ovipneumoniae* strain in a free-ranging desert bighorn sheep population. Animals (Basel) 12:1029 35454275 10.3390/ani12081029 PMC9028599

[19] Wang K, Liu X, Li Q, Wan K, Gao R, Han G, Li C, Xu M, Jia B, Shen X (2020) *MHC-DRB1* exon 2 polymorphism and its association with mycoplasma ovipneumonia resistance or susceptibility genotypes in sheep. J Genet 99:22 32366733

[20] Plowright RK, Manlove KR, Besser TE, Paez DJ, Andrews KR, Matthews PE, Waits LP, Hudson PJ, Cassirer EF (2017) Age-specific infectious period shapes dynamics of pneumonia in bighorn sheep. Ecol Lett 20:1325–1336 28871636 10.1111/ele.12829

[21] Johnson T, Jones K, Jacobson BT, Schearer J, Adams N, Thornton I, Mosdal C, Jones S, Jutila M, Rynda-Apple A, Besser T, Bimczok D (2022) Experimental infection of specific-pathogen-free domestic lambs with *Mycoplasma ovipneumoniae* causes asymptomatic colonization of the upper airways that is resistant to antibiotic treatment. Vet Microbiol 265:109334 35033769 10.1016/j.vetmic.2022.109334 PMC9109813

[22] Malmberg JL, Allen SE, Jennings-Gaines JE, Johnson M, Luukkonen KL, Robbins KM, Cornish TE, Smiley RA, Wagler BL, Gregory Z, Lutz D, Hnilicka P, Monteith KL, Edwards WH (2024) Pathology of chronic *Mycoplasma ovipneumoniae* carriers in a declining bighorn sheep (*Ovis canadensis*) population. J Wildl Dis 60:448–460 38329742 10.7589/JWD-D-23-00132

[23] Besser TE, Cassirer EF, Potter KA, VanderSchalie J, Fischer A, Knowles DP, Herndon DR, Rurangirwa FR, Weiser GC, Srikumaran S (2008) Association of *Mycoplasma ovipneumoniae* infection with population-limiting respiratory disease in free-ranging Rocky Mountain bighorn sheep (*Ovis canadensis canadensis*). J Clin Microbiol 46:423–430 18057131 10.1128/JCM.01931-07 PMC2238132

[24] Raghavan B, Erickson K, Kugadas A, Batra SA, Call DR, Davis MA, Foreyt WJ, Srikumaran S (2016) Role of carriers in the transmission of pneumonia in bighorn sheep (*Ovis canadensis*). Biol Open 5:745–755 27185269 10.1242/bio.018234 PMC4920194

[25] Garwood TJ, Lehman CP, Walsh DP, Cassirer EF, Besser TE, Jenks JA (2020) Removal of chronic *Mycoplasma ovipneumoniae* carrier ewes eliminates pneumonia in a bighorn sheep population. Ecol Evol 10:3491–3502 32274004 10.1002/ece3.6146 PMC7141075

[26] He X, Chen X, Wang H, Du G, Sun X (2023) Recent advances in respiratory immunization: a focus on COVID-19 vaccines. J Control Release 355:655–674 36787821 10.1016/j.jconrel.2023.02.011 PMC9937028

[27] Martin SA, Pence BD, Woods JA (2009) Exercise and respiratory tract viral infections. Exerc Sport Sci Rev 37:157–164 19955864 10.1097/JES.0b013e3181b7b57b PMC2803113

[28] Jones GE, Keir WA, Gilmour JS (1985) The pathogenicity of *Mycoplasma ovipneumoniae* and *Mycoplasma arginini* in ovine and caprine tracheal organ cultures. J Comp Pathol 95:477–487 4067018 10.1016/0021-9975(85)90018-0

[29] Ionas G, Mew AJ, Alley MR, Clarke JK, Robinson AJ, Marshall RB (1985) Colonisation of the respiratory tract of lambs by strains of *Mycoplasma ovipneumoniae*. Vet Microbiol 10:533–539 4095899 10.1016/0378-1135(85)90062-8

[30] Robinson E, Schulein C, Jacobson BT, Jones K, Sago J, Huber V, Jutila M, Bimczok D, Rynda-Apple A (2022) Pathophysiology of influenza D virus infection in specific-pathogen-free lambs with or without Prior *Mycoplasma ovipneumoniae* exposure. Viruses 14:1422 35891403 10.3390/v14071422 PMC9321583

[31] Coppock A (2022) randomizr: Easy-to-use tools for common forms of random assignment and sampling. R package version 0.22.0

[32] Passmore MR, Byrne L, Obonyo NG, See Hoe LE, Boon AC, Diab SD, Dunster KR, Bisht K, Tung JP, Fauzi MH, Narula M, Pedersen SE, Esguerra-Lallen A, Simonova G, Sultana A, Anstey CM, Shekar K, Maitland K, Suen JY, Fraser JF (2018) Inflammation and lung injury in an ovine model of fluid resuscitated endotoxemic shock. Respir Res 19:231 30466423 10.1186/s12931-018-0935-4 PMC6249903

[33] Shanthalingam S, Goldy A, Bavananthasivam J, Subramaniam R, Batra SA, Kugadas A, Raghavan B, Dassanayake RP, Jennings-Gaines JE, Killion HJ, Edwards WH, Ramsey JM, Anderson NJ, Wolff PL, Mansfield K, Bruning D, Srikumaran S (2014) PCR assay detects *Mannheimia haemolytica* in culture-negative pneumonic lung tissues of bighorn sheep (*Ovis canadensis*) from outbreaks in the western USA, 2009–2010. J Wildl Dis 50:1–10 24171569 10.7589/2012-09-225

[34] Bowen L, Manlove K, Roug A, Waters S, LaHue N, Wolff P (2022) Using transcriptomics to predict and visualize disease status in bighorn sheep (*Ovis canadensis*). Conserv Physiol 10:coac046 35795016 10.1093/conphys/coac046 PMC9252122

[35] R. Core Team (2024) R: a language and Environment for Statistical Computing

[36] Wickham H, Averick M, Bryan J, Chang W, McGowan LD, François R, Grolemund G, Hayes A, Henry L, Hester J, Kuhn M, Pedersen TL, Miller E, Bache SM, Müller K, Ooms J, Robinson D, Seidel DP, Spinu V, Takahashi K, Vaughan D, Wilke C, Woo K, Yutani H (2019) Welcome to the tidyverse. J Open Source Softw 4:1686

[37] Pinheiro JC, Bates DM (2000) Mixed-effects models in S and S-PLUS. Springer, New York

[38] Bates D, Mächler M, Bolker B, Walker S (2015) Fitting linear mixed-effects models using lme4. J Stat Softw 67:1–48

[39] Lüdecke D (2023) sjPlot: data visualization for statistics in social science. R package version 2.8.15

[40] Wickham H (2016) ggplot2: Elegant graphics for data analysis, 1^st^ edn. Springer, New York

[41] Arnold JB (2024) ggthemes: Extra themes, scales and Geoms for 'ggplot2'. R package version 5.1.0

[42] Annunziato F, Romagnani C, Romagnani S (2015) The 3 major types of innate and adaptive cell-mediated effector immunity. J Allergy Clin Immunol 135:626–635 25528359 10.1016/j.jaci.2014.11.001

[43] Besser TE, Cassirer EF, Potter KA, Foreyt WJ (2017) Exposure of bighorn sheep to domestic goats colonized with *Mycoplasma ovipneumoniae* induces sub-lethal pneumonia. PLoS One 12:e0178707 28591169 10.1371/journal.pone.0178707 PMC5462392

[44] Schielzeth H, Dingemanse NJ, Nakagawa S, Westneat DF, Allegue H, Teplitsky C, Réale D, Dochtermann NA, Garamszegi LZ, Araya-Ajoy YG (2020) Robustness of linear mixed-effects models to violations of distributional assumptions. Methods Ecol Evol 11:1141–1152

[45] Lindner T, Puck J, Verbeke A (2020) Misconceptions about multicollinearity in international business research: identification, consequences, and remedies. J Int Bus Stud 51:283–298

[46] Mousel MR, White SN, Herndon MK, Herndon DR, Taylor JB, Becker GM, Murdoch BM (2021) Genes involved in immune, gene translation and chromatin organization pathways associated with *Mycoplasma ovipneumoniae* presence in nasal secretions of domestic sheep. PLoS One 16:e0247209 34252097 10.1371/journal.pone.0247209 PMC8274911

[47] Jemski JV, Hetsko CM, Helms CM, Grizzard MB, Walker JS, Chanock RM (1977) Immunoprophylaxis of experimental *Mycoplasma pneumoniae* disease: effect of aerosol particle size and site of deposition of *M. pneumoniae* on the pattern of respiratory infection, disease, and immunity in hamsters. Infect Immun 16:93–98 873619 10.1128/iai.16.1.93-98.1977 PMC421493

[48] Halperin SA, Heifetz SA, Kasina A (1988) Experimental respiratory infection with Bordetella pertussis in mice: comparison of two methods. Clin Invest Med 11:297–303 3168352

[49] Grizzard MB, London WT, Sly DL, Murphy BR, James WD, Parnell WP, Chanock RM (1978) Experimental production of respiratory tract disease in cebus monkeys after intratracheal or intranasal infection with influenza A/Victoria/3/75 or influenza A/New Jersey/76 virus. Infect Immun 21:201–205 101457 10.1128/iai.21.1.201-205.1978 PMC421977

[50] Jones GE, Gilmour JS, Rae AG (1982) The effect of *Mycoplasma ovipneumoniae* and *Pasteurella haemolytica* on specific pathogen-free lambs. J Comp Pathol 92:261–266 6211466 10.1016/0021-9975(82)90084-6

[51] Jeyaseelan S, Sreevatsan S, Maheswaran SK (2002) Role of *Mannheimia haemolytica* leukotoxin in the pathogenesis of bovine pneumonic pasteurellosis. Anim Health Res Rev 3:69–82 12665107 10.1079/ahrr200242

[52] Lawrence PK, Dassanayake RP (2010) Ovis aries CR4 is involved in *Mannheimia haemolytica* leukotoxin-induced cytotoxicity. Vet Immunol Immunopathol 135:266–274 20060597 10.1016/j.vetimm.2009.12.007

[53] Niang M, Rosenbusch RF, DeBey MC, Niyo Y, Andrews JJ, Kaeberle ML (1998) Field isolates of *Mycoplasma ovipneumoniae* exhibit distinct cytopathic effects in ovine tracheal organ cultures. Zentralbl Veterinarmed A 45:29–40 9557125 10.1111/j.1439-0442.1998.tb00798.x

[54] Gilmour JS, Jones GE, Rae AG (1979) Experimental studies of chronic pneumonia of sheep. Comp Immunol Microbiol Infect Dis 1:285–293 318238 10.1016/0147-9571(79)90030-4

[55] Nunoya T, Omori T, Tomioka H, Umeda F, Suzuki T, Uetsuka K (2020) Intracellular localization of *Mycoplasma bovis* in the bronchiolar epithelium of experimentally infected calves. J Comp Pathol 176:14–18 32359627 10.1016/j.jcpa.2020.01.005

[56] Xiu F, Li X, Liu L, Xi Y, Yi X, Li Y, You X (2024) Mycoplasma invasion into host cells: an integrated model of infection strategy. Mol Microbiol 121:814–830 38293733 10.1111/mmi.15232

[57] Zhang YX, Li Y, Wang Y, Ren YF, Yang Y, Qi J, Yang H, Liang X, Zhang RF (2024) Prospective cohort study on the clinical significance of interferon-gamma, D-dimer, LDH, and CRP tests in children with severe mycoplasma pneumonia. Medicine (Baltimore) 103:e39665 39465799 10.1097/MD.0000000000039665 PMC11479529

[58] Li J, Chen C, Gao L, Wang L, Wang W, Zhang J, Gong Z, Wang J, Guo Y (2023) Analysis of histopathology and changes of major cytokines in the lesions caused by *Mycoplasma ovipneumoniae* infection. BMC Vet Res 19:273 38102682 10.1186/s12917-023-03829-4 PMC10722778

[59] Ziegler JC, Lahmers KK, Barrington GM, Parish SM, Kilzer K, Baker K, Besser TE (2014) Safety and immunogenicity of a *Mycoplasma ovipneumoniae* bacterin for domestic sheep (*Ovis aries*). PLoS One 9:e95698 24752006 10.1371/journal.pone.0095698 PMC3994082

[60] Yang F, Dao X, Rodriguez-Palacios A, Feng X, Tang C, Yang X, Yue H (2014) A real-time PCR for detection and quantification of *Mycoplasma ovipneumoniae*. J Vet Med Sci 76:1631–1634 25649947 10.1292/jvms.14-0094 PMC4300380

