# Supplementary material for: Pathogen delivery route impacts disease severity in experimental Mycoplasma ovipneumoniae infection of domestic lambs
Source: Vet Res. 2025 Jan 13;56:10. doi: 10.1186/s13567-024-01439-y (PMC11731165; doi:10.1186/s13567-024-01439-y)
Supplement: Supplementary file 1 — Additional file 1: Rubric for health scoring. Scoring rubric for lamb health during challenge experiment, based on Johnson et al. [21]. [file 13567_2024_1439_MOESM1_ESM.docx]

**Additional file 1: Scoring rubric for lamb health, based on Johnson et al. [21].**

| **Parameters** | **0** | **1** | **2** | **3** | **4** | **5** |
| --- | --- | --- | --- | --- | --- | --- |
| **Behavior** | Bright, alert, responsive | Small change in attitude/ behavior | Less active, visibly appears ill | Ill but still responsive | Lethargic | Unresponsive |
| **Appetite** | Ate well | Ate most of the ration | Ate ¾ feed | Ate ½ feed | Ate < ½ feed | Not eating |
| **Respiratory Signs** | No nasal or ocular discharge, no cough, normal respirations | Panting/ increased respiratory rate, or slight clear nasal/ocular discharge | Clear nasal or ocular discharge, or coughed 1-5 times | Moderate ocular or nasal discharge or frequent coughing | Mucous, nasal, or ocular discharge, coughing, lamb visibly unwell | Discharge, coughing, wheezing, labored breathing |
| **Medication** | No treatments | Electrolytes | NSAID | Antibiotic | NSAID and antibiotics | Multiple NSAIDs and antibiotics |
