# Supplementary figures and images for: Pathogen delivery route impacts disease severity in experimental Mycoplasma ovipneumoniae infection of domestic lambs
Source: Vet Res. 2025 Jan 13;56:10. doi: 10.1186/s13567-024-01439-y (PMC11731165; doi:10.1186/s13567-024-01439-y)

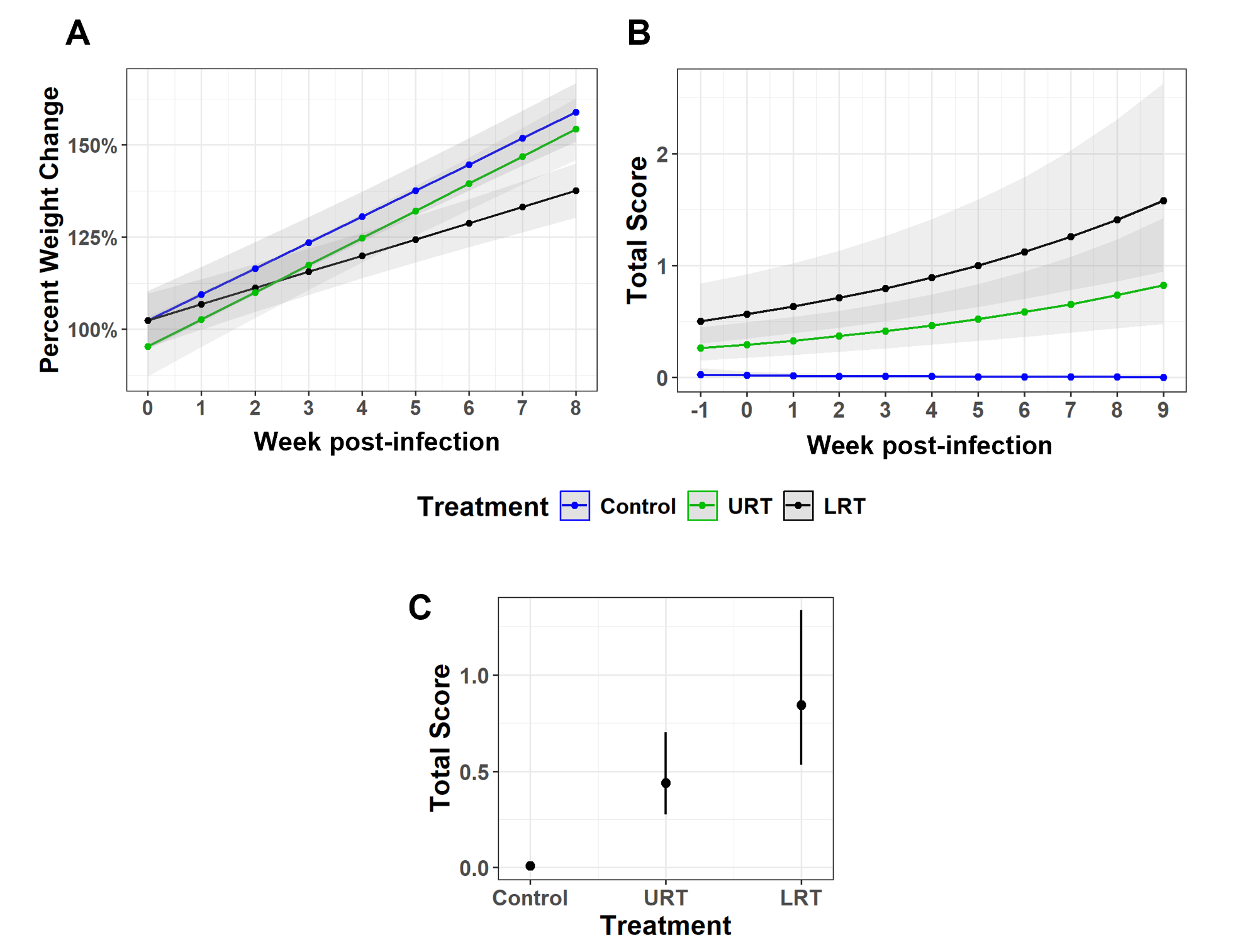

Supplement: Supplementary file 2 — Additional file 2: Linear mixed modeling of lamb weight development and health. (A) The predicted percent weight change effect is shown for the treatments across the weeks while holding sex and age at infection constant. The grey shaded area corresponds to the 95% confidence interval. (B) The predicted total score effect is shown for each treatment group across the weeks post-infection holding sex and age at infection constant. The grey shaded area corresponds to the 95% confidence interval. (C) The predicted total score effect is shown for just the treatment groups while holding week, sex, and age at infection constant, the lines correspond to the 95% confidence interval. [file 13567_2024_1439_MOESM2_ESM.tif]

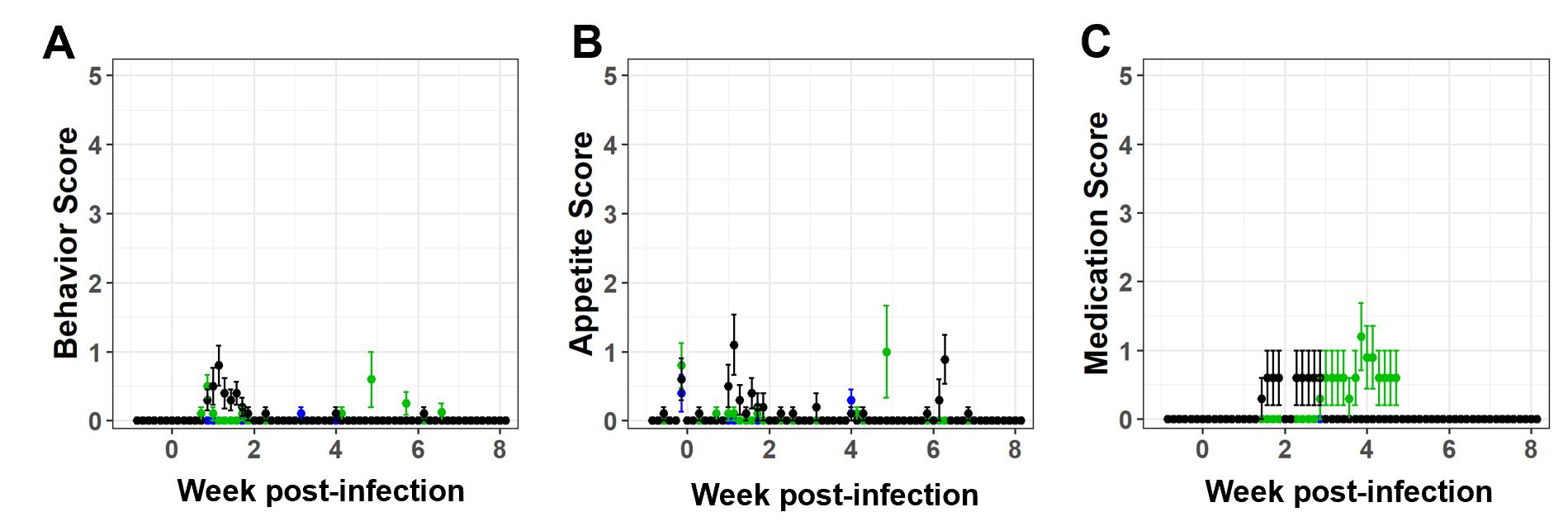

Supplement: Supplementary file 3 — Additional file 3: Health scores for behavior, appetite, and administered medications, and body temperatures. All lambs were screened twice daily for changes in (A) behavior, and (B) appetite. (C) Administered medications were also recorded (see Additional file 1 for scoring rubric). Daily scores were the sum of the two individual measurements. Graphs show mean ± SD of five lambs per group. [file 13567_2024_1439_MOESM3_ESM.tif]

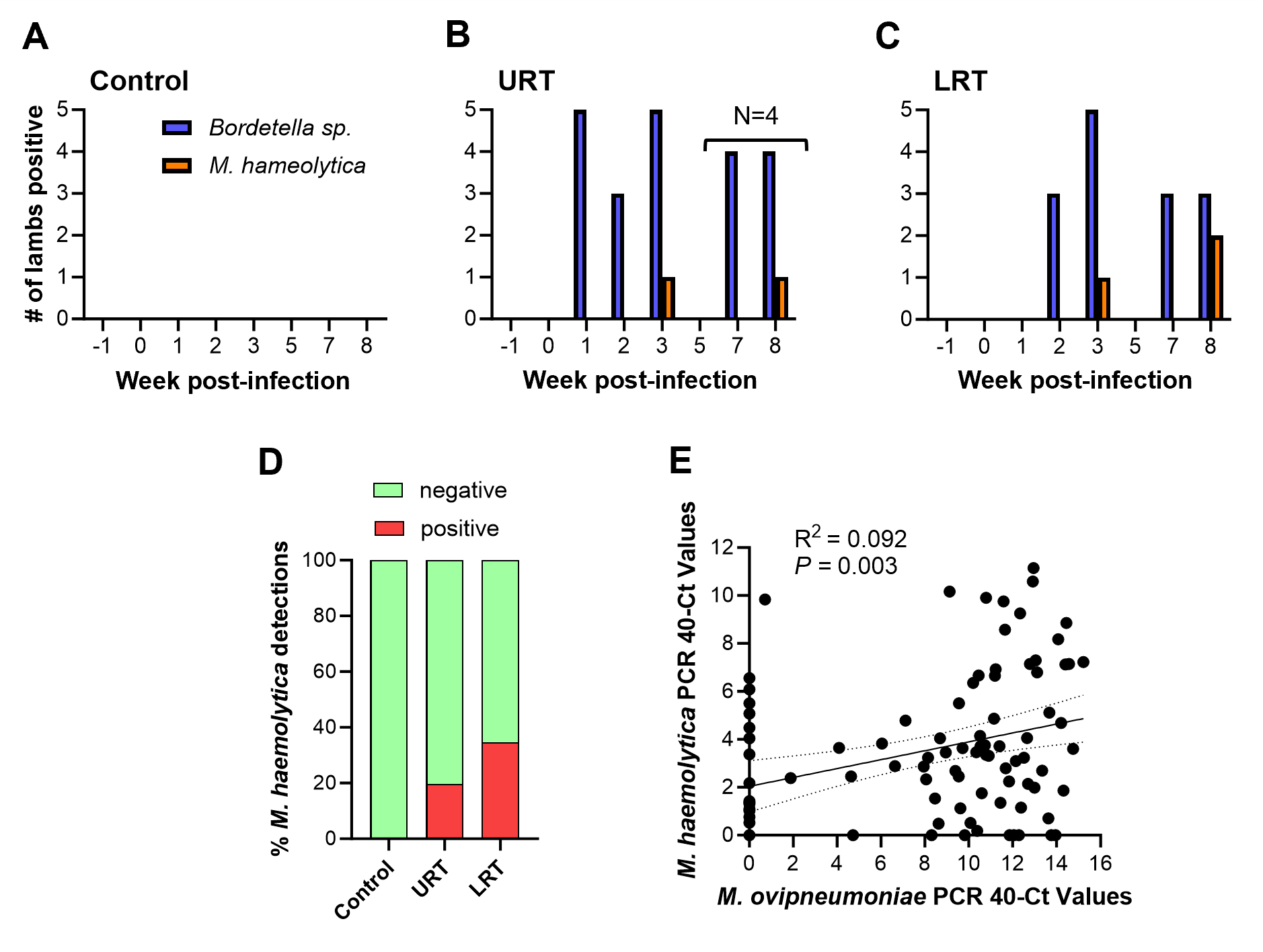

Supplement: Supplementary file 4 — Additional file 4: Identification of Pasteurellacea in M. ovipneumoniae-infected lambs. Routine bacteriological analysis of nasal swab samples collected throughout the study from lambs in (A) the control group, (B) the upper respiratory tract (URT) infection group, and (C) the lower respiratory tract (LRT) infection group for the presence of Pasteurellacea was performed at the Washington Animal Diseases Diagnostic Laboratory. (D) Percentage of Mannheimia haemolytica positive nasal swab samples out of all samples in lambs from the three experimental groups. (E) Weak correlation between Ct values from M. ovipneumoniae and M. haemolytica PCRs across all animals from the URT and LRT groups and all time points post inoculation. Data were analyzed by simple linear regression analysis. [file 13567_2024_1439_MOESM4_ESM.tif]
